# Integration of Genome-Wide Computation DRE Search, AhR ChIP-chip and Gene Expression Analyses of TCDD-Elicited Responses in the Mouse Liver

**DOI:** 10.1186/1471-2164-12-365

**Published:** 2011-07-15

**Authors:** Edward Dere, Raymond Lo, Trine Celius, Jason Matthews, Timothy R Zacharewski

**Affiliations:** 1 Department of Biochemistry & Molecular Biology, Michigan State University, East Lansing, MI, 48824, USA; 2 Department of Pharmacology & Toxicology, University of Toronto, Toronto, Ontario, M5S 1A8, Canada; 3 Center for Integrative Toxicology, Michigan State University, East Lansing, MI, 48824, USA

## Abstract

**Background:**

The aryl hydrocarbon receptor (AhR) is a ligand-activated transcription factor (TF) that mediates responses to 2,3,7,8-tetrachlorodibenzo-*p*-dioxin (TCDD). Integration of TCDD-induced genome-wide AhR enrichment, differential gene expression and computational dioxin response element (DRE) analyses further elucidate the hepatic AhR regulatory network.

**Results:**

Global ChIP-chip and gene expression analyses were performed on hepatic tissue from immature ovariectomized mice orally gavaged with 30 μg/kg TCDD. ChIP-chip analysis identified 14,446 and 974 AhR enriched regions (1% false discovery rate) at 2 and 24 hrs, respectively. Enrichment density was greatest in the proximal promoter, and more specifically, within ± 1.5 kb of a transcriptional start site (TSS). AhR enrichment also occurred distal to a TSS (e.g. intergenic DNA and 3' UTR), extending the potential gene expression regulatory roles of the AhR. Although TF binding site analyses identified over-represented DRE sequences within enriched regions, approximately 50% of all AhR enriched regions lacked a DRE core (5'-GCGTG-3'). Microarray analysis identified 1,896 number of TCDD-responsive genes (|fold change| ≥ 1.5, P1(t) > 0.999). Integrating this gene expression data with our ChIP-chip and DRE analyses only identified 625 differentially expressed genes that involved an AhR interaction at a DRE. Functional annotation analysis of differentially regulated genes associated with AhR enrichment identified overrepresented processes related to fatty acid and lipid metabolism and transport, and xenobiotic metabolism, which are consistent with TCDD-elicited steatosis in the mouse liver.

**Conclusions:**

Details of the AhR regulatory network have been expanded to include AhR-DNA interactions within intragenic and intergenic genomic regions. Moreover, the AhR can interact with DNA independent of a DRE core suggesting there are alternative mechanisms of AhR-mediated gene regulation.

## Background

The aryl hydrocarbon receptor (AhR) is a ligand activated transcription factor (TF) belonging to the basic-helix-loop-helix-PAS (bHLH-PAS) family of proteins that serve as environmental sensors [1]. 2,3,7,8-Tetrachlorodibenzo-*p*-dioxin (TCDD) is the prototypical AhR ligand, a ubiquitous environmental contaminant that elicits diverse species-specific effects, including tumor promotion, teratogenesis, hepatotoxicity, modulation of endocrine systems, immunotoxicity and enzyme induction [2,3]. These effects result from alterations in gene expression mediated by the AhR [4]. Several studies have demonstrated the requirement for the AhR in mediating TCDD-elicited responses. For example, mice carrying low-affinity AhR alleles are less susceptible to the effects elicited by TCDD [5]. Additionally, AhR-null mice fail to induce responses typically observed following treatment with TCDD and related compounds [6].

TCDD binding to the cytosolic AhR results in a conformational change and translocation to the nucleus. The activated AhR complex heterodimerizes with the aryl hydrocarbon nuclear translocator (ARNT), another bHLH-PAS family member, and binds dioxin response elements (DREs) containing the substitution intolerant 5'-GCGTG-3' core sequence to regulate changes in gene expression [4,7]. Computational searches for all DRE cores in the human, mouse and rat genome identified the highest density of DREs proximal to a transcriptional start site (TSS) [8]. However, a significant number of DRE cores and putative functional DREs have been identified in distal regions within non-coding intergenic segments of the genome. It has been proposed that enrichments for other TFs on outlying regions may be functionally relevant through tertiary looping of genomic DNA and/or via protein tethering mechanisms [9].

The role of specific transcriptional regulators has been studied on a gene-by-gene basis, primarily focusing on regions proximal to the TSS. However, the coupling of chromatin immunoprecipitation with either genomic tiling microarrays (ChIP-chip) or next-generation sequencing (ChIP-seq) has facilitated genome-wide analysis of protein-DNA interactions for a variety of receptors [10-16], TFs [17-20] and components of the basal transcriptional machinery [10,21,22]. Genome-wide location analyses further suggest that TF binding at *cis*-regulatory enhancers in intergenic DNA regions of the genome may also have functional significance [10,17,23,24].

Several studies have investigated AhR-mediated gene expression responses using various technologies [25-30]. Although AhR-DNA interactions have primarily focused on the regulation of *CYP1A1 *[4,31], recent global ChIP studies have extended our knowledge of AhR-DNA interactions by examining promoter region binding profiles using *in vitro *and *in vivo *models [32-35] (Lo *et al*., in submission). Our study provides a comprehensive analysis by examining TCDD-induced AhR binding across the entire mouse genome. In addition, we examined AhR binding within chromosomes, intragenic and intergenic DNA regions, and in specific genic regions (i.e., 10 kb upstream of a TSS, 5' and 3' untranslated regions [UTRs], coding sequence [CDS]). Global AhR enrichment data are also integrated with computational DRE core analysis [8], and complementary whole-genome gene expression profiling to provide a more comprehensive evaluation of the hepatic AhR regulatory network elicited by TCDD.

## Results

### Identification and Characterization of TCDD-Elicited AhR Enrichment

**Figure 1:**
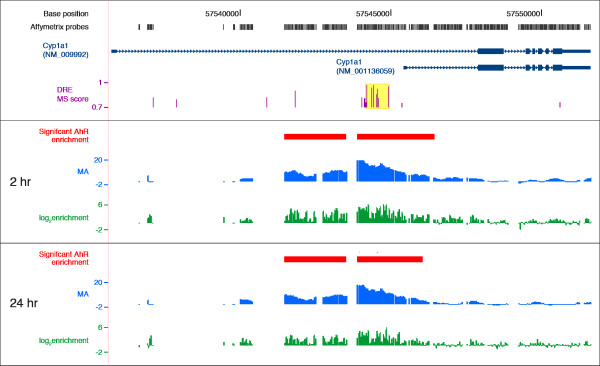
**Summary of AhR enrichment within *Cyp1a1 *genic region at 2 and 24 hrs**. *Cyp1a1 *is represented by two RefSeq sequences (NM_009992 and NM_001136059, dark blue tracks) that have different TSSs (dark blue box at far left). The rectangles and lines represent exons and introns, respectively, and the UTRs are depicted as the thinner rectangles. Arrowhead direction indicates the orientation of the gene. The grey boxes above represent the Affymetrix 2.0R mouse tiling array probe locations across the *Cyp1a1 *genic regions. The location and matrix similarity (MS) scores of the consensus DREs are represented by the purple histogram. The highlighted yellow box identifies *bona fide *functional DREs (matrix similarity (MS) score ≥ 0.8473) involved in AhR-mediated *Cyp1a1 *gene expression. The red boxes identify regions of significant AhR enrichments (FDR < 0.01) based on the moving average (MA) profile by TileMap. The green histogram plots the log_2 _fold enrichment values for each individual probe.

In order to identify regions of AhR enrichment induced by TCDD across the genome, ChIP-chip assays were performed using hepatic tissue from immature ovariectomized mice orally gavaged with 30 μg/kg TCDD for 2 and 24 hrs. CisGenome [36] analysis identified 22,502 and 12,677 enriched regions at 2 and 24 hrs, respectively. Applying a conservative FDR of 0.01 resulted in 14,446 and 974 significant AhR enriched regions at 2 and 24 hrs, respectively (Additional Files 1 and 2 provides a complete list of enriched regions). Ligand activation of the AhR *in vivo *triggers its own rapid degradation and causing a significant reduction of AhR levels [37,38]. This is reflected in the significantly lower number of TCDD-induced AhR enriched regions at 24 hrs as compared to 2 hrs. The distribution, location and enrichment values for each tiled probes across the *Cyp1a1 *gene (represented by RefSeq sequences NM_009992 and NM_001136059) are summarized in Figure 1. MA value plots visualize the profile of the enriched region and log_2 _fold-enrichment values for each probe are also illustrated (Figure 1). Note that the probes are unevenly tiled throughout the genome, resulting in gaps in genome coverage that may coincide with DRE core locations that may affect AhR enriched region identification. For example, two enriched regions were associated with *Cyp1a1 *(Figure 1, red bars). However, the MA plots for 2 and 24 hrs suggest that there is only one large region of enrichment divided into two as a result of the uneven tiling. Consequently, uneven tiling and the lack of tiling in regions that contain DREs may affect the estimated number of AhR enriched regions.

**Figure 2:**
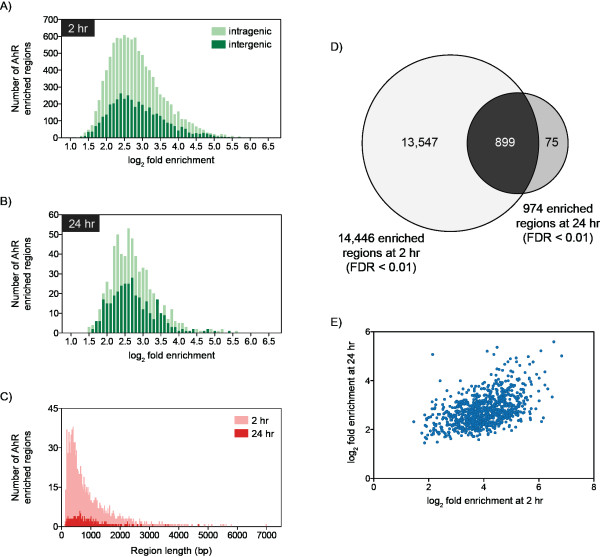
**Characterization of TCDD-induced AhR enriched regions at 2 and 24 hrs (FDR < 0.01)**. Frequency analysis of enriched regions relative to log_2 _fold enrichment at 2 hr (**A**) and 24 hr (**B**) illustrating enrichment values in intragenic (light green) and intergenic (dark green) DNA regions. Distribution of enriched regions relative to region width (**C**) at 2 hrs (light red) and 24 hrs (dark red) identified 90.5% of enriched sites were ≤ 1,500 bp. Comparison of AhR enriched regions at 2 and 24 hrs identified 899 overlapping regions (**D**). Analysis of the fold enrichment values for the 899 overlapping regions at 2 and 24 hrs identified a positive correlation (two-tailed P-value < 0.0001, Pearson correlation coefficient = 0.4853; **E**).

Genomic regions with significant AhR enrichment were mapped to intragenic (10 kb upstream of a TSS plus the transcribed gene of mature RefSeq sequences) and non-coding intergenic regions (Table 1; Additional File 3). Most regions were enriched 5.7-fold with values ranging from 1.7- to 111.4-fold (Figures 2A-B). Enriched regions varied in width from 108 to 6,990 bp (Figure 2C) with 90.5% spanning ≤ 1,500 bp. There was no correlation between fold enrichment and region width (data not shown). Of the 974 significantly enriched regions at 24 h 899 of them overlapped with a 2 hr enriched region (Figure 2D), consistent with reports of constant shuttling of the AhR between the nucleus and cytoplasm [39], and AhR promoter occupancy of targeted genes in untreated cells [34]. Relaxing the FDR to 0.05 increased the overlap to 906, while reducing the number of 24 hr specific enriched regions to 68. Comparable overlaps were identified in promoter-specific ChIP-chip studies of TCDD-induced AhR enrichment at 2 and 24 hrs in the livers of intact C57BL/6 mice, which identified 1,397 number of genes with 403 overlap (Lo *et al*., in submission). Further analysis of the 899 enriched regions found that the fold enrichment values from both time points were positively correlated (Pearson correlation coefficient = 0.4853, two-tailed p-value < 0.0001; Figure 2E).

**Table 1:** Distribution and density analysis of TCDD-induced AhR enriched regions^a^ in the mouse genome.

					Genic Region^c^			
					
		Genome	Intergenic DNA^b^	Intragenic DNA^b^	10kb upstream	5' UTR	CDS	3' UTR
**2 hr**	AhR enrichment	14,446	4,163	10,283	4,601	2,569	7,499	225
	Enrichment density^d^	5.44	2.62	9.64	18.65	17.29	7.21	7.29

**24 hr**	AhR enrichment	974	344	630	306	132	507	9
	Enrichment density^d^	0.37	0.22	0.59	1.24	0.89	0.49	0.29

^a ^AhR enriched regions with a FDR < 0.01

^b ^intergenic, intragenic and gene regions are defined as previously described in Additional File 1

^c ^regions are defined using the genomic locations in the refGene database from the UCSC Genome Browser

^d ^density calculated per million base pairs

**Figure 3:**
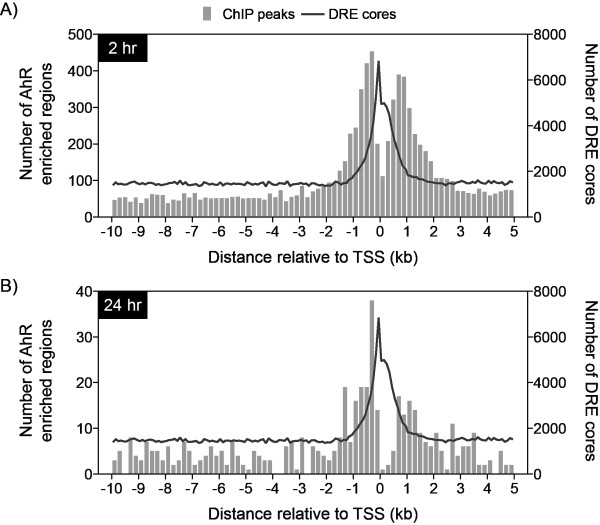
**TCDD-induced AhR enrichment (FDR < 0.01) densities in the proximal promoter (10 kb upstream and 5 kb downstream of a TSS) at 2 hrs (A) and 24 hrs (B)**. The bars represent the number of enriched regions in each 200 bp window. The number of DRE cores in 100 bp non-overlapping windows is superimposed (line) illustrating the overlap between AhR enriched regions and DRE cores in the proximal promoter region.

Although only 40% of the mouse genome consists of intragenic DNA, 71.8% and 64.7% of all sites with significant AhR enrichment at 2 hrs and 24 hrs, respectively, were within this region. The density of AhR enrichment (per million base pairs [Mbp]) was calculated across the entire genome in order to consider the cumulative intergenic and intragenic DNA region lengths (Table 1). Genome and chromosomal analyses (Additional Files 4 and 5) revealed increased enrichment within intragenic regions compared to non-coding intergenic regions further illustrating a bias for gene encoding regions. However, these values may be inflated due to incomplete probe coverage in the intergenic regions and sequence gaps in the genome. Specific analysis of the 10 kb upstream, 5' and 3' UTRs and CDS regions revealed the highest density of AhR enrichment was proximal to the TSS (Table 1 and Additional Files 4 and 5). AhR enrichment density was greatest within ± 1.5 kb at 2 and 24 hrs (Figures 3A-B), coinciding with proximal promoter DRE core densities [8] and RNA polymerase II binding at the TSSs [10]. Interestingly, there is a notable cleft in AhR enrichment 200 bp directly upstream and downstream of the TSSs, possibly to accommodate general transcription machinery. Both global and proximal promoter density analyses illustrate TCDD-induced AhR enrichments are more prominent in regions directly associated with a gene. Nevertheless, there are a significant number of distally located enrichment sites that may also be functionally relevant.

### Confirmation of AhR ChIP-chip Enrichment Analysis

**Figure 4:**
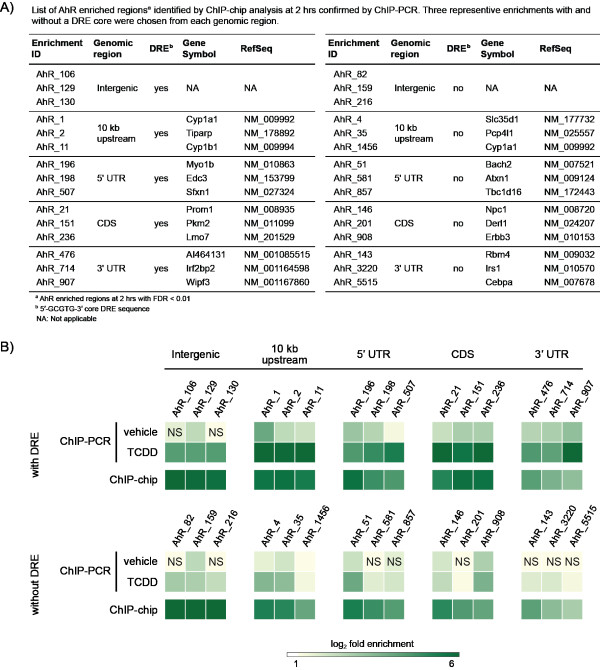
**Confirmation of hepatic TCDD-induced AhR enrichment identified by ChIP-chip analysis (FDR < 0.01) at 2 hrs by ChIP-PCR**. Selected regions were chosen for verification based on position relative to a TSS, ChIP-chip fold enrichment and the presence or lack of a DRE core within the region of enrichment (**A**). Immunoprecipitated DNA was measured by QRTPCR and AhR enrichment was calculated as fold induction above IgG controls. The color intensity of each box represents the mean value of three independent replicates. NS = not significant compared to IgG controls (p < 0.05). 2 hr ChIP-chip enrichment values are provided in Additional File 1.

Selected regions of AhR enrichment identified by ChIP-chip analysis at 2 hrs were confirmed by ChIP-PCR (Figure 4). Three representative ChIP-chip enrichments from each genomic region (intergenic, 10 kb upstream of a TSS, 5' UTR, CDS and 3' UTR) were selected to validate AhR enrichments with and without a DRE core at different positions relative to the TSS. ChIP-PCR and ChIP-chip analysis of DRE containing regions exhibited similar levels of AhR enrichment relative to IgG_TCDD _controls and were significantly greater than vehicle controls relative to IgG_vehicle_. AhR enriched regions without the DRE core were also verified, further demonstrating that the AhR can interact with DNA independent of a DRE core, but does not eliminate the possibility of AhR interaction through DNA looping or protein tethering. Interestingly, the fold enrichment values for regions without the DRE core were consistently lower than those with a DRE core, suggesting AhR interactions are stronger in regions containing a DRE.

### DRE Analysis of AhR Enriched Regions

**Figure 5:**
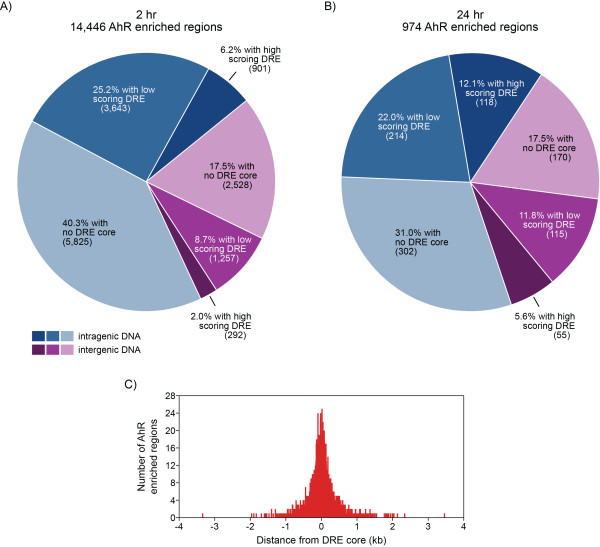
**Mapping TCDD-induced AhR enriched regions (FDR < 0.01) with DRE locations**. Regions of enrichment identified in the intergenic (purple) and intragenic (blue) DNA regions of the genome at 2 hrs (**A**) and 24 hrs (**B**) were searched for high scoring (putative functional) DRE sequences (matrix similarity score ≥ 0.8473; dark blue and dark purple segments) and low scoring DRE sequences (matrix similarity score < 0.8473; mid blue and mid purple segments) using a position weight matrix developed from *bona fide *functional DREs [8]. Light blue and light purple segments represent regions with no DRE core sequence. A total of 6,595 enriched regions (6,093 at 2 hrs and 502 at 24 hrs) contained at least one DRE core (5'-GCGTG-3'). 50% of these regions were within 135 bp of a DRE core (based on the location of maximum enrichment within the enriched region; **C**).

TCDD-elicited changes in gene expression are mediated through AhR signaling via binding to the substitution intolerant DRE core sequence (5'-GCGTG-3'). Overlaying TCDD-induced AhR enrichment with DRE core locations throughout the mouse genome [8] identified 57.8% and 48.5% of the enriched regions did not contain a DRE core regions at 2 and 24 hrs, respectively (Table 2 and Figures 5A-B). Other promoter-specific ChIP-chip studies have also reported DRE cores in ~50% of the AhR enriched regions [33,35]. The remaining enriched regions possessed at least one and as many as 16 DRE cores (Table 2). AhR enriched regions with or without a DRE core exhibited similar widths and levels of enrichment.

**Table 2:** Distribution of DRE cores in AhR enriched regions^a^.

Number of DRE cores^b^	Number of AhR enriched regions	
	
	2 hr	24 hr
0	8,353	472
1	3,705	289
2	1,372	121
3	544	46
4	223	16
5	109	12
6	67	7
7	25	5
8	15	0
9	11	3
10	7	1
11	5	0
12	3	1
13	0	0
14	3	1
15	3	0
16	1	0

**Total**	**14,446**	**974**

^a ^AhR enriched regions with a FDR < 0.01

^b ^5'-GCGTG-3' core sequence

Matrix similarity (MS) scores have been calculated for each 19 bp DRE sequence within the mouse genome using a position weight matrix (PWM) constructed from *bona fide *functional DREs [8]. Of the 6,595 significant AhR enriched regions containing a DRE core (6,093 from 2 hr and 502 from 24 hr), 90.7% were within 500 bp of a DRE core (i.e. distance of maximum enrichment within the region to an underlying DRE core) with half of these positions located within 135 bp of a DRE core. However, only 8.3% and 17.8% of the AhR enriched regions at 2 and 24 hrs, respectively, possessed a putative functional (high scoring) DRE sequence (MS score ≥ 0.8473) suggesting the AhR may bind other degenerate sequence elements.

AhR binding to an alternate response element (5'-CATGN_6_C[T|A]TG-3') has also been reported [40,41]. Of the 8,353 and 472 enriched regions at 2 and 24 hrs, respectively, that did not contain a DRE core, 482 and 237, respectively, contained the alternate DRE sequence (5.8% and 50.2%, respectively). The higher incidence of AhR enriched regions at 24 hrs containing the alternate response element may represent tertiary AhR binding sites resulting from conformational changes and crowding of the promoter with the general transcription machinery [42,43].

### Transcription Factor Binding Site Over-Representation Analysis

Significantly AhR enriched regions were computationally analyzed for over-represented response elements for known TF binding site (TFBS) families using RegionMiner (Genomatix). DREs as well other sites for early growth response (EGR), E2F, nuclear respiratory factor 1 (NRF1), nuclear receptor subfamily 2 factors (NR2F/COUP-TF) and peroxisome proliferator-activated receptor (PPAR) were over-represented within AhR enriched regions (Table 3; complete list of over-represented TFBS are provided in Additional Files 6 and 7). Many of these TF sites were enriched proximally to a DRE core (i.e. within 10-50 bp; Additional File 8) suggesting possible interactions. Studies have previously reported interactions between AhR and many of these TFs [34,44,45]. For example, AhR complexes with EGR-1 following treatment of human HUVEC cells with high glucose concentrations [45]. In addition, AhR aggregates with E2F1 to inhibit E2F1-induced apoptosis [46]. AhR also directly interacts with COUP-TF to repress ER-mediated gene expression [47].

**Table 3:** Significantly over-represent transcription factor module families in TCDD-induced AhR enriched regions^a^.

TF Module Family	Module Description	2 hr AhR enriched regions				24 hr AhR enriched regions			
			
		# of matches	Expected # of matches	Over-representaion^b^	Z-Score	# of matches	Expected # of matches	Over-representaion^b^	Z-Score
AHR	AhR-ARNT heterodimer	9,447	4,278.30	2.21	79.03	851	297.44	2.86	32.07
SP1	GC-Box factors SP1/GC	19,356	12,839.04	1.51	57.54	1,346	892.61	1.51	15.17
HIF	Hypoxia inducible factor, bHLH/PAS protein family	8,763	4,841.13	1.81	56.37	569	336.57	1.69	12.64
E2F	E2F-Myc activator/cell cycle regulator	18,247	12,444.88	1.47	52.04	1,266	865.21	1.46	13.62
ZBP	Zinc binding protein factors	25,542	18,518.20	1.38	51.65	1,739	1,287.45	1.35	12.58
NRF1	Nuclear respiratory factor 1	3.475	1,494.81	2.32	51.21	252	103.92	2.42	14.48
ZF5	ZF5 POZ domain zinc finger	3,156	1,442.92	2.19	45.09	205	100.32	2.04	10.40
NF1	Nuclear factor 1	13,047	8,876.90	1.47	44.27	886	617.15	1.44	10.81
NR2F	Nuclear receptor subfamily 2 factors	44,774	36,390.64	1.23	44.02	3,180	2,530.00	1.26	12.93
EGR	EGR/nerve growth factor included protein c	22,224	16,794.62	1.32	41.92	1,541	1,167.62	1.32	11.
PPAR	Peroxisome proliferator-activated receptor	24,808	19,035.70	1.30	41.87	1,752	1,323.43	1.32	11.78
RXR	RXR heterodimer binding sites	41,027	33,441.00	1.23	41.54	2,932	2,324.93	1.26	12.60
WHN	Winged helix binding sites	3,030	1,477.23	2.05	40.39	206	102.70	2.01	10.14

^a ^AhR enriched regions with a FDR < 0.01

^b ^total number of matches in enriched regions/expected number of matches in genome

Complete list of over-represented TF module families are provided in Additional Files 6 and 7

### *De Novo *Motif Analysis

**Figure 6:**
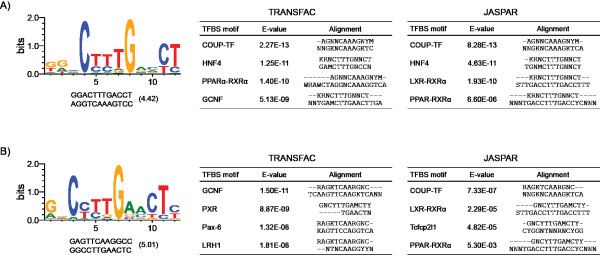
***De novo *motif analysis of intragenic (**A**) and intergenic (**B**) AhR enriched regions lacking a DRE core**. The non-repetitive over-represented motifs from each region are shown with their consensus and reverse complement sequence, and the Gibbs motif sampler score. Over-represented motifs were associated with specific TFBSs in JASPAR and TRANSFAC based on the consensus sequence alignments and E-value scores.

Approximately 50% of enriched regions lacked the DRE core sequence (Figures 5A-B) suggesting AhR interacts with DNA using alternate strategies. *De novo *motif analysis of these regions using the Gibbs motif sampler in CisGenome identified over-representation of comparable repetitive elements in both the intergenic and intragenic DNA regions (Additional File 9). Comparison of over-represented non-repetitive motifs to existing TF binding motifs in JASPAR and TRANSFAC [48,49] using STAMP [50] identified similarities to COUP-TF, hepatocyte nuclear factor 4 (HNF4), liver receptor homolog 1 (LRH1/NR5A2) and PPAR binding sites (Figure 6). Interestingly, COUP-TF and HNF4 belong to the NR2F family identified in the TFBS over-representation analysis of all AhR enriched regions (Table 3). The presence of these binding motifs in non-DRE containing regions of AhR enrichment further suggests that AhR-DNA interactions occur through a tethering mechanism involving other TFs or by tertiary looping of DNA.

### Gene Level Analysis of AhR Enrichment

**Figure 7:**
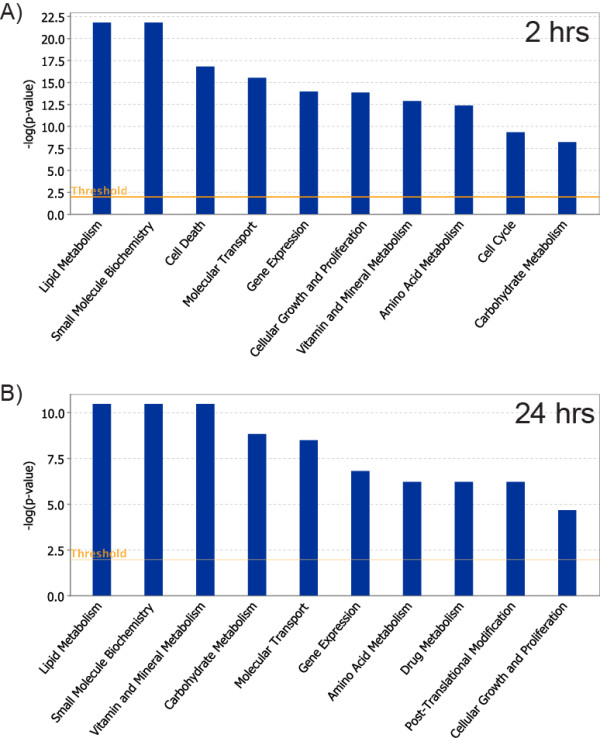
**Molecular and cellar functions over-represented by genes associated with significant AhR enrichment (FDR < 0.01) containing a DRE core**. The 4,544 and 332 unique genes with AhR enrichment with a DRE core at 2 hrs (**A**) and 24 hrs (**B**), respectively, were analyzed using Ingenuity Pathway Analysis for enriched biological functions using Fisher's Exact Test (p < 0.01; orange line). The blue bars represent the log Odds value calculated from the p-value of each functional group.

**Figure 8:**
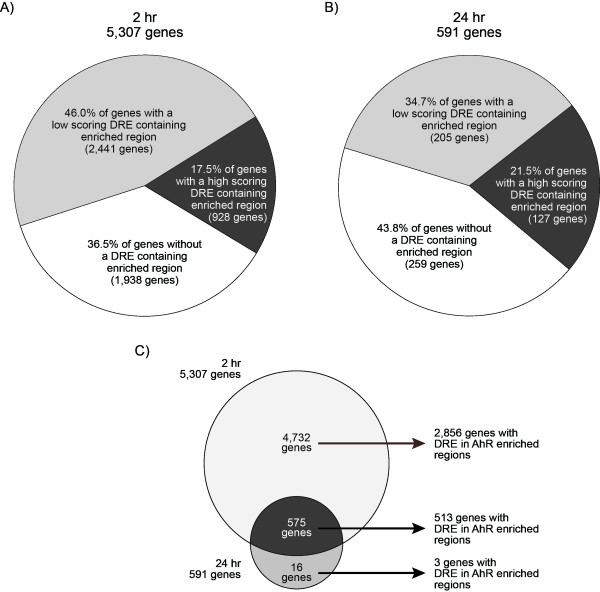
**Mapping TCDD-induced AhR enriched regions (FDR < 0.01) and DRE analysis to genes**. The 10,283 and 660 AhR enrichments within the intragenic DNA regions at 2 and 24 hrs (blue shaded areas in Figures 5A-B) mapped to 5,307 (**A**) and 591 (**B**) distinct genes based on the refGene data from the UCSC Genome Browser. These genes were searched for the presence of high (matrix similarity score (MS) ≥ 0.8473; dark grey areas) and low (MS score < 0.84731; light grey areas) scoring DRE sequences, and the absence of a DRE core (white areas) within the region of AhR enrichment. Comparing 2 and 24 hrs data identified 575 overlapping genes with AhR enrichment and 513 of these genes contained a DRE core within the region of enrichment (**C**).

Of the 10,369 enrichments identified in the intragenic DNA regions, 43.8% (4,544/10,369) contained a DRE core at 2 hrs, and 52.4% (332/634) at 24 hrs (Figure 5, areas shaded blue). These intragenic AhR enriched regions mapped to 5,307 and 591 unique genes at 2 and 24 hrs, respectively (AhR targeted genes are provided as gene annotated enriched regions in Additional Files 1 and 2). Molecular and cellular functional analysis using Ingenuity Pathway Analysis (IPA) found these genes to be associated with lipid and carbohydrate metabolism, small molecule biochemistry, cell cycle and gene expression based on a Fisher's Exact Test p-value < 0.01 (Figure 7; Additional Files 10 and 11 list the most significant over represented biological functions at 2 and 24 hrs). Furthermore, 63.5 and 56.2% of the genes associated with AhR enrichment at 2 and 24 hrs, respectively, contained a DRE core within the region of enrichment (Figure 8). The higher percentage of genes containing a DRE core compared to enriched regions with a DRE core is due to multiple regions of AhR enrichment associated with a single gene (as illustrated for *Cyp1a1 *in Figure 1). The remaining genes (36.5% and 54.8% at 2 and 24 hrs, respectively) with significant AhR enrichment were targeted independently of a DRE core.

At both 2 and 24 hrs, 575 genes had AhR enrichment, with 513 possessing DRE cores in the AhR enriched region (Figure 8C). Only 16 genes exhibited AhR enrichment solely at 24 hrs, with three containing a DRE core. In contrast, 4,732 genes possessed significant AhR enrichment with 60.4% (2,856) containing a DRE core within the region of enrichment at 2 hrs. Due to the large overlap of enriched regions at 2 and 24 hrs, the remaining analysis focuses predominantly on the AhR enrichment at 2 hr.

### Comparison of Transcriptional Responses with AhR Enrichment

**Figure 9:**
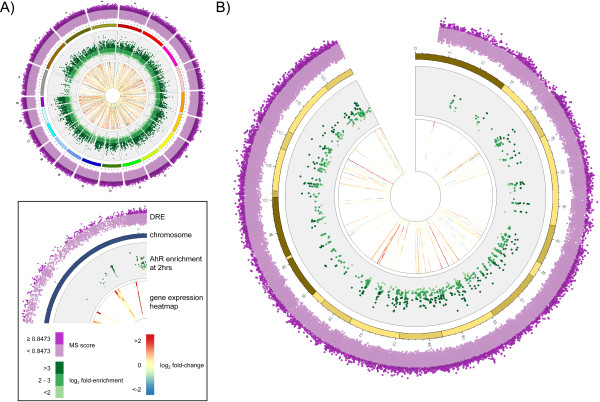
**Circos plots integrating DRE analysis, AhR enrichment (2 hrs; FDR < 0.01) and heatmaps for hepatic differential gene expression responses (|fold change| ≥ 1.5 and P1(t) > 0.999) induced by TCDD across the genome (A) and chromosome 9 (B)**. The inset legend image provides information represented by each data ring. DRE matrix similarity (MS) scores and AhR enrichment values increase radially outward. The time points for the gene expression heatmaps also increase radial outward. The arc of each heatmap wedge maps directly to the location of the gene in the genome. The arc length is proportional to the length of the transcribed region. Circos plots for the other chromosomes are provided in Additional File 12.

Gene expression analysis at 2, 4, 8, 12, 18, 24, 72, and 168 hrs identified 1,896 unique differentially expressed genes (|fold change| ≥ 1.5 and P1(t) > 0.999) at one or more time points. Of the 1,896 TCDD-responsive genes, 900 genes (47.5%) possessed significant AhR enrichment within the intragenic region (10 kb upstream of the TSS to the end of the transcript). Moreover, of the 900 genes exhibiting AhR enrichment at 2 hrs, 625 contained a DRE core sequence, suggesting these responses are AhR-mediated. The remaining 275 differentially expressed genes were not associated with a AhR enriched region containing a DRE core, and may be secondary responses. In order to concisely visualize the integration of the DRE, ChIP-chip and gene expression analyses, Circos plots were generated for the genome and individual chromosomes (Figure 9 and Additional File 12). The plots further illustrate the diversity in AhR enrichment locations in relation to the genomic position of dysregulated genes. Further analysis of the responsive genes found that most were induced by TCDD (Table 4) at all time points. Greater than 82% of the induced genes at 2 or 4 hrs had significant AhR enrichment, and more than 62% of them contained at least one DRE core suggesting that regulation is DRE-dependent fashion. In contrast, only 35% of the 691 genes induced at 168 hrs, exhibited AhR enrichment with 26% possessing a DRE core suggesting that these are secondary gene expression responses. Interestingly, down-regulated genes associated with AhR enrichment were relatively consistent across all time points. Approximately one third of the down-regulated genes appear to be AhR regulated with DRE involvement.

**Table 4:** Distribution and AhR enrichment and DRE analysis of differentially expressed genes elicited by TCDD.

			2 hr	4 hr	8 hr	12 hr	18 hr	24 hr	72 hr	168 hr
**Number of differentially expressed genes^a^**	Up-regulated	Total	68	255	341	218	236	287	267	719
		With AhR enrichment^b^	55	200	202	148	172	186	164	243
		With AhR enrichment^b ^+ DRE core^c^	47	168	171	126	146	156	135	181
	
	Down-regulated	Total	18	233	116	168	105	237	261	218
		With AhR enrichment^b^	10	123	76	102	59	131	137	123
		With AhR enrichment^b ^+ DRE core^c^	6	79	49	63	30	72	79	70

^a ^|fold-change| ≥ 1.5 and P1(t) > 0.999

^b ^AhR enriched regions at 2 hrs with FDR < 0.01

^c ^5'-GCGTG-3' core sequence within AhR enriched region

**Figure 10:**
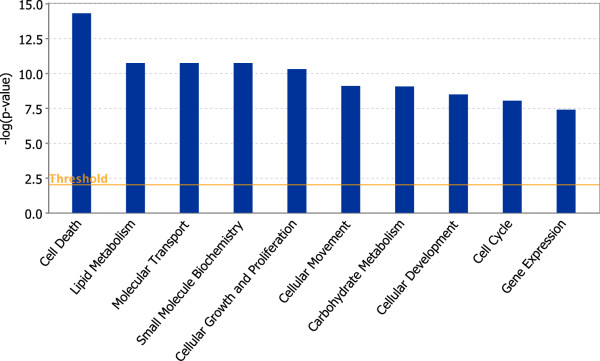
**Molecular and cellar functions over-represented by differentially regulated genes (|fold change| ≥ 1.5, P1(t) > 0.999) associated with significant AhR enrichment (FDR < 0.01) at 2 hrs**. The 900 differentially regulated genes with AhR enrichment were analyzed using Ingenuity Pathway Analysis for enriched biological functions using Fisher's Exact Test (p < 0.01; orange line). The blue bars represent the log Odds value calculated from the p-value of each functional group.

Functional analysis of the 900 differentially expressed genes associated with AhR enrichment was performed using DAVID [51]. The most over-represented functions were associated with lipid metabolic processes (enrichment score of 7.34, Table 5), consistent with the induced fatty liver phenotype [52,53]. IPA analysis of these genes also identified lipid metabolism as an enriched molecular and cellular function (Fisher's Exact Test p-value < 0.01; Figure 10; Additional File 13 provides a list of the most significant biological functions). In addition, *de novo *motif analysis (Figure 6) identified binding sites for TFs associated with lipid metabolism and transport. The induction of AhR regulated xenobiotic enzymes, such as cytochrome P450s, glutathione S-transferases (Gsts) and UDP-glucuronosyltransferases (Ugts), hallmarks of TCDD exposure, were also identified as an enriched cluster (enrichment score of 3.54).

**Table 5:** Functional enrichment analysis of differently regulated^a ^genes with AhR enrichment^b ^using DAVID.

Category	Team	Gene Count	Fold enrichment	P-value
**Enrichment Score: 7.34**				
GOTERM_BP_3	GO:0006629 ~ lipid metabolic process	76	2.23	5.53E-11
GOTERM_BP_3	GO:0044255 ~ cellular lipid metabolic process	53	2.23	7.80E-08
GOTERM_BP_3	GO:0008610 ~ lipid biosynthetic process	32	2.30	2.28E-05
**Enrichment Score:4.12**				
GOTERM_BP_3	GO:0048523 ~ negative regulation of cellular process	94	1.60	4.46E-06
GOTERM_BP_3	GO:0048519 ~ negative regulation of biological process	101	1.54	7.83E-06
GOTERM_BP_3	GO:0031324 ~ negative regulation of cellular metabolic process	45	1.85	8.99E-05
GOTERM_BP_3	GO:0051172 ~ negative regulation of nitrogen compound metabolic process	39	1.96	9.68E-05
GOTERM_BP_3	GO:0009890 ~ negative regulation of biosynthetic process	40	1.86	2.33E-04
GOTERM_BP_3	GO:0009892 ~ negative regulation of metabolic process	46	1.74	3.20E-04
GOTERM_BP_3	GO:0010605 ~ negative regulation of macromolecule metabolic process	43	1.72	6.88E-04
**Enrichment Score: 3.54**				
GOTERM_BP_3	GO:0009410 ~ response to xenobiotic stimulus	8	10.59	3.54E-06
GOTERM_BP_3	GO:0006805 ~ xenobiotic metabolic process	7	11.59	1.12E-05
GOTERM_BP_3	GO:0018894 ~ dibenzo-p-dioxin metabolic process	3	19.86	7.31E-03
GOTERM_BP_3	GO:0009404 ~ toxin metabolic process	3	11.92	2.28E-02
**Enrichment Score: 2.70**				
GOTERM_BP_3	GO:0051272 ~ positive regulation of call motion	9	4.58	6.00E-04
GOTERM_BP_3	GO:0051270 ~ regulation of cell motion	15	2.84	7.55E-04
GOTERM_BP_3	GO:0040017 ~ Positive regulation of locomotion	9	4.26	1.01E-03
GOTERM_BP_3	GO:0030334 ~ regulation of cell migration	13	2.87	1.76E-03
GOTERM_BP_3	GO:0040012 ~ regulation of locomotion	13	2.39	7.93E-03
GOTERM_BP_3	GO:0040012 ~ regulation of localization	32	1.61	9.36E-03
**Enrichment Score: 2.55**				
GOTERM_BP_3	GO:0048518 ~ positive regulation of biological process	106	1.44	7.45E-05
GOTERM_BP_3	GO:0048522 ~ positive regulation of cellular process	94	1.45	2.03E-04
GOTERM_BP_3	GO:0009893 ~ positive regulation of metabolic process	51	1.53	2.64E-03
GOTERM_BP_3	GO:0010604 ~ positive regulation of macromolecule metabolic process	48	1.54	3.02E-03
GOTERM_BP_3	GO:0031325 ~ positive regulation of cellular	47	1.48	6.67E-03
GOTERM_BP_3	GO:0009891 ~ positive regulation of biosynthetic	38	1.38	3.96E-02
GOTERM_BP_3	GO:0031325 ~ positive regulation of nitrogen compound metabolic process	36	1.39	4.40E-02
**Enrichment Score: 2.44**				
GOTERM_BP_3	GO:0005996 ~ monosaccharide metabolic process	21	2.27	9.50E-04
GOTERM_BP_3	GO:0005975 ~ carbohydrate metabolic process	37	1.67	2.63E-03
GOTERM_BP_3	GO:0016051 ~ carbohydrate biosynthetic process	11	2.70	7.19E-03
GOTERM_BP_3	GO:0044262 ~ cellular carbohydrate metabolic process	27	1.70	9.34E-03

^a ^|fold-change| ≥ 1.5 and P1(t) >0.999 at one or more time points

^b ^AhR enriched regions at 2 hrs with FDR < 0.01

Although AhR mediates the expression of enzymes involved in xenobiotic metabolizing enzymes, including NADP(H) dehydrogenase, quinone 1 (*Nqo1*) and UDP-glucose dehydrogenase (*Ugdh*) as well as several Ugt and Gst isoforms, they are also regulated by nuclear factor, erythroid derived 2, like 2 (Nrf2) via antioxidant response elements in response to oxidative stress [54,55]. Recent studies with AhR and Nrf2 null mice report that TCDD induction of *Nqo1 *is AhR and Nrf2 dependent [56]. Furthermore, specific Ugt and Gst isoforms induced by TCDD require Nrf2. Collectively, these responses are referred to as the "TCDD-inducible AhR-Nrf2 gene battery." ChIP-chip and gene expression analysis indicates that *Nqo1*, *Gstm1*, *Gstm2*, *Ugdh *and *Nrf2 *induction is associated with AhR enrichment. Although supportive of the Nrf2-dependency model, these data do not distinguish if these are secondary responses mediated by Nrf2 alone, or involve an AhR-Nrf2 interaction. In contrast, *Gsta1 *and *Ugt2b35 *induction occurred independently of AhR enrichment, suggesting they may only be dependent on Nrf2 [56].

Immune cell accumulation following a single acute dose of TCDD at 168 hrs is presumed to be a secondary response to hepatic injury or fatty acid accumulation [52,53]. DAVID analysis of genes induced at 168 hrs identified multiple over-represented immune-related clusters (enrichment scores > 2). However, several of the genes including complement component 1, q subcomponent, beta polypeptide (*C1qb*), CD36 antigen (*Cd36*), complement component 4A (C4a) and interferon regulatory factor 8 (Irf8), did not exhibit accompanying AhR enrichment within their intragenic region (10 kb upstream of the TSS to the end of the 3' UTR). Only 26 out of 105 differentially regulated genes in the enriched immune clusters exhibited AhR enrichment. Collectively, these data suggest that gene expression associated with immune function is a consequence of immune cell infiltration into the liver.

## Discussion

This study further elucidates the role of the AhR in mediating the hepatic effects of TCDD in C57BL6 mice. Recent studies have mapped AhR binding using promoter-focused ChIP-chip arrays and found that ~50% of the AhR enriched regions were devoid of the DRE core [32-34]. The lack of a DRE core in regions of AhR enrichment was also reported in a AhR genome-wide ChIP-chip study performed in mouse CH12.LX cells [57]. ChIP-seq experiments for other TFs have also demonstrated enrichment in remote genome regions, which may serve important regulatory roles [10,11,14,17]. Collectively these data suggest the AhR uses different mechanisms to regulate gene expression. Moreover, the integration of genome-wide *in silico *DRE search, with *de novo *motif analysis and TCDD-elicited hepatic temporal gene expression data has further elucidated the hepatic AhR gene regulatory network.

ChIP-chip analysis identified 14,446 TCDD-induced AhR regions at 2 hrs and 974 regions at 24 hrs, consistent with the rapid nuclear export and subsequent degradation of the AhR following TCDD activation [37]. Approximately half of these regions were within intragenic regions (10 kb upstream of a TSS to the end of the 3' UTR). Furthermore, 25% of these enriched regions at 2 hrs and 19% at 24 hrs were within 2 kb of a TSS, indicating that a large subset of AhR enrichment occurs adjacent to a TSS. Unlike other studies that report a normal distribution of TF binding centered around the TSS [15,58-60], the AhR density profile exhibited a cleft immediately adjacent to the TSS, possibly to accommodate recruited transcriptional machinery.

Although most AhR enrichment regions are intragenic, a significant number are located in distal intergenic regions (i.e. 4,163 of 14,446 at 2 hrs and 344 of 974 at 24 hrs). Studies with the ER, p53 and forkhead box protein A1 [10,11,14,17] suggest distal TF binding may have distinct regulatory roles. Binding proximal to the TSS is presumed to stabilize the general transcriptional machinery, while distal binding regulates transcription by a looping mechanism or by altering chromatin structure [9,61,62]. Consequently, AhR binding outside of the proximal promoter region may have important regulatory roles that remain largely uninvestigated.

Comparing AhR enriched regions with DRE cores revealed that their intergenic, intragenic and genic (10 kb upstream, UTRs, and CDS) density distributions were similar. The greatest density of AhR enrichment associated with a DRE core occurred within the proximal promoter. Both exhibited comparable distribution profiles except for the cleft in enrichment at the TSS. The decrease in AhR enrichment at the TSS coincides with RNA polymerase II binding at the TSSs [10] of transcriptionally responsive genes. Although TCDD-elicited differential gene expression is thought to be mediated by the substitution intolerant DRE core sequence (5'-GCGTG-3'), only ~50% of the AhR enriched regions contained a DRE core, consistent with findings in other promoter targeted AhR ChIP-chip studies [33,35] (Lo *et al*., in submission). Moreover, relatively few alternative AhR response elements (5'-CATGN_6_C[T|A]TG-3') [40,41] were identified in AhR enriched regions lacking a DRE core sequence. Enrichment in regions lacking DRE cores provides additional evidence of AhR-DNA interactions that don not involve the basic bHLH domain [63], such as tethering to other DNA interacting TFs and/or tertiary interactions with looping DNA.

Integration of gene expression, ChIP-chip, and DRE distribution data suggests that ~35% of all differentially expressed hepatic genes are mediated by direct AhR binding to a DRE. Consequently, 65% of the gene expression responses elicited by TCDD do not involve direct AhR binding to a DRE. However, TF binding analyses based on tiling arrays is limited by the extent of probe coverage (Figure 1). Genomic regions lacking probe coverage may falsely inflate the number of DRE-absent AhR enriched regions, thus underestimating the number of AhR regulated genes involving a DRE. Furthermore, the analyses may not be exhaustive due to the technical limitations of ChIP-chip assay coupled with the conservative FDR threshold used to identify statistically significant signals, which may have excluded some positive signals. These limitations of the technology could be addressed in ChIP-seq experiments, which have greater resolution and sensitivity [64,65]. The shorter sequence reads would improve resolution, but may also identify fewer regions containing a DRE. The higher sensitivity of ChIP-seq could also identify additional regions of AhR enrichment. ChIP-seq studies could also confirm AhR binding in these genomic regions in either a DRE-dependent or -independent manner.

TCDD induces hepatic vacuolization and lipid accumulation with differential gene expression associated with fatty acid metabolism and transport [25,53]. Independent functional annotation analysis of differentially expressed genes with significant AhR enrichment using DAVID and IPA identified over-represented processes related to fatty acid and lipid metabolism. Computational analysis also identified over-represented binding motifs for TFs involved in the regulation of lipid and cholesterol metabolism, including sites for HNF4, LXR, PXR, PPAR and COUP-TF. COUP-TF is a potent repressor that antagonizes transcriptional responses mediated by other nuclear receptors including HNF4, PPAR, ER, RAR and VDR [66]. For example, COUP-TF antagonizes HNF4α-mediated responses by binding HNF4α response elements [67-71]. Furthermore, AhR interactions with COUP-TF repress ER-mediated gene expression responses [47]. Therefore, AhR interactions with COUP-TF may regulate lipid and fatty acid metabolism by blocking HNF4α target gene expression (Figure 10A). Coincidentally, the HNF4 binding motif is over represented within AhR enriched regions lacking a DRE core.

Consistent with this proposed mechanism, several HNF4α regulated genes, including *Cyp7a1 *and *Gck*, exhibited AhR enrichment and were repressed by TCDD. Cyp7a1 is the rate-limiting enzyme in the bile acid biosynthetic pathway that converts cholesterol into bile acids. Transgenic mice over-expressing Cyp7a1 are protected from high-fat diet induced obesity, fatty liver and insulin resistance [72]. Moreover, a genetic deficiency of Cyp7a1 in humans results in hyperlipidemia [73]. Gck phosphorylates glucose in the initial step of glycolysis. Mutations in *Gck *that reduce kinase activity are associated with insulin resistance and maturity onset diabetes of young 2 (MODY2) in humans [74-76]. Furthermore, mice over-expressing Gck are resistant to MODY2 [77]. The down-regulation of *Cyp7a1 *and *Gck*, possibly due to AhR - COUP-TF interactions at HNF4α response elements, is consistent TCDD-induced hepatic lipid accumulation in mice. Interestingly, TCDD exposure has been linked to diabetes and metabolic syndrome in humans [78-84]. Studies examining AhR-COUP-TF interactions and their effects on HNF4 target gene expression are being investigated further.

## Conclusion

This study identified the genome-wide locations of TCDD-induced hepatic AhR enrichment *in vivo *and incorporates DRE distribution and differential gene expression data to further elucidate the hepatic AhR regulatory network. In addition to identifying interactions in regions associated with genes, AhR enrichment in distal non-coding intergenic regions was characterized. The functional significance of these distal interactions is unknown but intergenic binding has been reported for other TFs, and warrants further investigation. Moreover, only ~50% of all AhR enriched regions involved a DRE, suggesting that indirect AhR binding to DNA plays a significant role in the AhR regulatory network.

## Methods

### Animal Handling and Treatment

Hepatic tissue samples from immature female ovariectomized C57BL/6 mice obtained from a previous study [53] were used for both ChIP assays at 2 and 24 hrs, and gene expression analyses across all time points. Briefly, mice were orally gavaged with 30 μg/kg TCDD and sacrificed by cervical dislocation at 2, 4, 8, 12, 18, 24, 72 or 168 hrs postdose. Tissues were removed, weighed, and multiple samples (~100 mg each) were flash frozen in liquid nitrogen and stored at -80°C until further use.

### Chromatin Immunoprecipitation (ChIP) and ChIP-chip Experiments

ChIP assays were performed as previously described [33] with the following changes. Approximately 100 mg of mouse liver was homogenized in 1% formaldehyde and incubated for 10 min at room temperature. Tissue homogenate was centrifuged at 10,000 RPM for 3 min at 4°C. Pellet was washed in ice-cold PBS, centrifuged, and resuspended in 900 μL of TSEI (20 mM Tris-HCl [pH 8.0], 150 mM NaCl, 2 mM EDTA, 1% Triton X-100, 0.1% sodium dodecyl sulfate) + 1× Protease Inhibitor Cocktail (Sigma, St. Louis, MO). Samples were sonicated 12 times for 10 s each time at 25% amplitude using a Branson 450 sonifier. Supernatant was transferred to fresh microcentrifuge tubes and incubated with rabbit IgG (5 μg; Sigma) and anti-AhR (5 μg; SA-210, Biomol) overnight at 4°C under gentle agitation. ChIP samples were washed and the DNA was isolated as previously described [33]. For ChIP-chip experiments, immunoprecipitated DNA isolated following immunoprecipitation with anti-AhR of liver extracts from TCDD-treated mice was linearly amplified using a whole genome amplification kit according to the manufacturer's instructions (Sigma). Linearly amplified DNA (7.5 μg) was fragmented by limited DNAseI digestion and hybridized to Affymetrix GeneChip^® ^mouse 2.0R tiling arrays (Affymetrix, Santa Clara, CA) as previously described [33]. The hybridization and washing steps were performed according to the manufacturer's protocol at the Centre for Applied Genomics (Toronto, Canada). Data were normalized and analyzed using CisGenome and mapped against mouse genome version mm9 [36]. Enriched regions with a false discovery rate (FDR) of 1.0% (0.01) were determined by comparing triplicate samples of AhR_TCDD _to triplicate IgG_TCDD _using a moving average (MA) approach with default settings in TileMap v2 [85]. Regions were merged if the gap between them was < 300 bp and the number of probes failing to reach the cut-off was < 5. Regions were discarded if they were < 120 bp or did not contain at least 5 continuous probes above the cut-off. ChIPed DNA was purified using the PCR purification kit from BioBasic Inc. (Markham, ON) and quantified using quantitative real-time PCR (QRTPCR) (KAPA SYBR Fast qPCR Master Mix; KAPA Biosystems, Toronto, ON) (ChIP-PCR). Fold enrichment values were calculated relative to IgG controls. ChIP-PCR primer sequences are provided in Additional File 14.

### ChIP-chip Location Analysis

The mouse genomic assembly (mm9) and associated annotation within the refGene and refLink databases were downloaded from the UCSC Genome Browser [86]. Individual segments of a gene region (i.e. the 10 kb sequence upstream of a TSS, the 5' and 3' UTRs and the CDS) for each mature gene encoding reference sequence (RefSeqs with NM prefixed identifiers) were determined using the genomic coordinates within the refGene databases (Additional File 3). Intragenic DNA regions within the genomes were computationally identified by merging overlapping gene regions (Additional File 3) from both strands of the genome, and the DNA between adjacent intragenic regions are defined as the non-transcribed intergenic DNA regions (Additional File 3). AhR enrichment densities were calculated based on the number of significant enriched regions occurring in an interrogated region (e.g. intergenic DNA region or 5' UTR) divided by the total sum of the region length. Gene annotation associated with each RefSeq sequence was derived from the refLink database in the UCSC Genome Browser.

### Transcription Factor Motif Analysis

The locations of AhR enrichment were compared against 5'-GCGTG-3' DRE core sequence locations in the mouse genome [8]. Identification of TF motifs over-represented in regions containing a DRE core were performed using the default parameter settings in RegionMiner, a program within the Genomatix suite of applications http://www.genomatix.de that contains an extensive database of TF binding motifs. Identified module families and individual matrices with z-scores > 3 were considered significant [87]. *De novo *motif discovery was performed using the Gibbs motif sampler in CisGenome on AhR regions of enrichment sequences not containing a DRE. Matrices for over-represented motifs were compared to existing TF binding motifs in JASPAR and TRANSFAC [48,49] using STAMP [50].

### Comparison with Microarray Gene Expression

Results from the ChIP-chip and DRE analysis were integrated with whole-genome gene expression profiling data from mice orally gavaged with 30 μg/kg TCDD using 4 × 44 k whole-genome oligonucleotide arrays from Agilent Technologies (Santa Clara, CA) [8]. The genomic locations of the differentially responsive genes (|fold change| ≥ 1.5 and P1(t) > 0.999) were obtained for each RefSeq sequence associated with the gene from the refGene database in the UCSC Genome Browser. Circos plots [88] were generated to visualize the locations of DRE cores, regions of AhR enrichment and temporal heatmaps of temporal gene expression responses.

### Functional Annotation and Pathway Analysis

Functional annotation clustering of Gene Ontology (GO) terms for genes associated with significant AhR enrichment was performed using DAVID (Database for Annotation, Visualization, and Integrated Discovery) [51]. In addition, the regions were analyzed using Ingenuity Pathway Analysis (IPA; http://www.ingenuity.com/) to identify over-represented molecular and cellular functions based on the Fisher's Exact Test p-value < 0.01.

## Authors' contributions

ED performed the computational analyses and integration of the ChIP-chip data with the computational DRE and microarray gene expression analyses, and the initial preparation of the manuscript. JM optimized and performed the ChIP experiments, and normalized the ChIP-chip data. TL and RL validated the ChIP-chip results with ChIP-PCR from regions identified by ED. TRZ oversaw the completion of the study. All the authors have given final approval of the version to be published.

## Supplementary Material

Additional file 1 **Genomic location, gene annotation and enrichment values of significant (FDR < 0.01) TCDD-induced AhR enrichment at 2 hrs**. Detailed results of the AhR ChIP-chip analysis that include the genomic location and TCDD-induced enrichment values, and the gene annotation of enrichment peaks located within the 10 kb upstream and transcribed region of a gene. Click here for file

Additional file 2 **Genomic location, gene annotation and enrichment values of significant (FDR < 0.01) TCDD-induced AhR enrichment at 24 hrs**. Detailed results of the AhR ChIP-chip analysis that include the genomic location and TCDD-induced enrichment values, and the gene annotation of enrichment peaks located within the 10 kb upstream and transcribed region of a gene. Click here for file

Additional file 3 **Definitions of various genomic regions used to map regions of AhR enrichment**. **A) **Genomic locations from the UCSC Genome Browser refGene database were used to obtain sequences for 10 kb region upstream of the TSS, the 5' and 3' UTRs, and the CDS of every known human, mouse and rat RefSeq sequence. A gene region is defined as the sequence spanning the region 10 kb upstream of a TSS through to the end of the 3' UTR. **B) **Intragenic DNA regions in a genome were determined by combining the non-overlapping gene regions. For example, gene regions of tissue specific isoforms of a gene that have different TSS positions were merged to determine the longest spanning range (genes C & C' and genes E & E'). Additionally, overlapping genes on both strands of the genome were also merged (genes B + E + E'). Non-transcribed DNA segments that span the regions between adjacent intragenic regions are defined as the intergenic DNA regions. Click here for file

Additional file 4 **TCDD-induced AhR enrichment (FDR < 0.01) density across the mouse genome at 2 hrs**. The density of significant AhR enrichment (per Mbp) at 2 hrs were calculated for each of the defined genomic regions across the individual chromosomes. Click here for file

Additional file 5 **TCDD-induced AhR enrichment (FDR < 0.01) density across the mouse genome at 24 hrs**. The density of significant AhR enrichment (per Mbp) at 24 hrs were calculated for each of the defined genomic regions across the individual chromosomes. Click here for file

Additional file 6 **Transcription factor binding site analysis of significant TCDD-induced AhR enrichment (FDR < 0.01) at 2 hrs**. DNA sequences for the regions of significant AhR enrichment at 2 hrs were analyzed for transcription factor (TF) binding site motif over-representation using RegionMiner. The results list the TF matrices and their corresponding over-representation and z-score value. Click here for file

Additional file 7 **Transcription factor binding site analysis of significant TCDD-induced AhR enrichment (FDR < 0.01) at 24 hrs**. DNA sequences for the regions of significant AhR enrichment at 24 hrs were analyzed for transcription factor (TF) binding site motif over-representation using RegionMiner. The results list the TF matrices and their corresponding over-representation and z-score value. Click here for file

Additional file 8 **Over-representation of transcription factor binding motifs located proximally (10-50 bp) of a DRE in a significantly AhR enriched region (FDR < 0.01)**. DNA sequences for the regions of significant AhR enrichment at 2 and 24 hrs possessing a DRE core sequence (5'-GCGTG-3') were analyzed for transcription factor (TF) binding site motif over-representation using RegionMiner. The results list the TF matrices and their corresponding over-representation and z-score value. Click here for file

Additional file 9 **Repetitive sequence elements identified in the *de novo *motif analysis of significant intragenic and intergenic AhR enriched regions (FDR < 0.01) lacking a DRE core**. The repetitive over-represented motifs from each region are shown with their consensus and reverse complement sequence, and the Gibbs motif sampler score. Click here for file

Additional file 10 **Pathway analysis of genes associated with DRE-containing regions of AhR enrichment (FDR < 0.01) at 2 hrs**. List of the most significant Bio-Functions (p < 0.01) identified using Ingenuity Pathway Analysis for the genes associated with a significant AhR enriched region (FDR < 0.01) containing a DRE core (5'-GCGTG-3') at 2 hrs. Click here for file

Additional file 11 **Pathway analysis of genes associated with DRE-containing regions of AhR enrichment (FDR < 0.01) at 24 hrs**. List of the most significant Bio-Functions (p < 0.01) identified using Ingenuity Pathway Analysis for the genes associated with a significant AhR enriched region (FDR < 0.01) containing a DRE core (5'-GCGTG-3') at 24 hrs. Click here for file

Additional file 12 **Circos plots integrating DRE analysis, AhR enrichment (2 hrs; FDR < 0.01) and heatmaps for hepatic differential gene expression responses (|fold change| ≥ 1.5 and P1(t) > 0.999) induced by TCDD across the genome**. Circos plots illustrate the ideograms for each individual chromosome and the entire genome and integrate the results of the DRE, ChIP-chip and gene expression analyses. Click here for file

Additional file 13 **Pathway analysis of differentially regulated genes (|fold change| ≥ 1.5 and P1(t) > 0.999) associated with regions of AhR enrichment (FDR < 0.01) at 2 hrs**. List of the most significant Bio-Functions (p < 0.01) identified using Ingenuity Pathway Analysis for TCDD-elicited gene expression responses (|fold change| ≥ 1.5 and P1(t) > 0.999) associated with a significant AhR enriched region (FDR < 0.01) containing a DRE core (5'-GCGTG-3') at 24 hrs. Click here for file

Additional file 14 **Primer sequences used to verify 2 hr ChIP-chip responses**. QRTPCR primers used to verify AhR enriched regions isolated from the 2 hr ChIP-chip. Click here for file
