# Supplementary figures and images for: Integration of Genome-Wide Computation DRE Search, AhR ChIP-chip and Gene Expression Analyses of TCDD-Elicited Responses in the Mouse Liver
Source: BMC Genomics. 2011 Jul 15;12:365. doi: 10.1186/1471-2164-12-365 (PMC3160422; doi:10.1186/1471-2164-12-365)

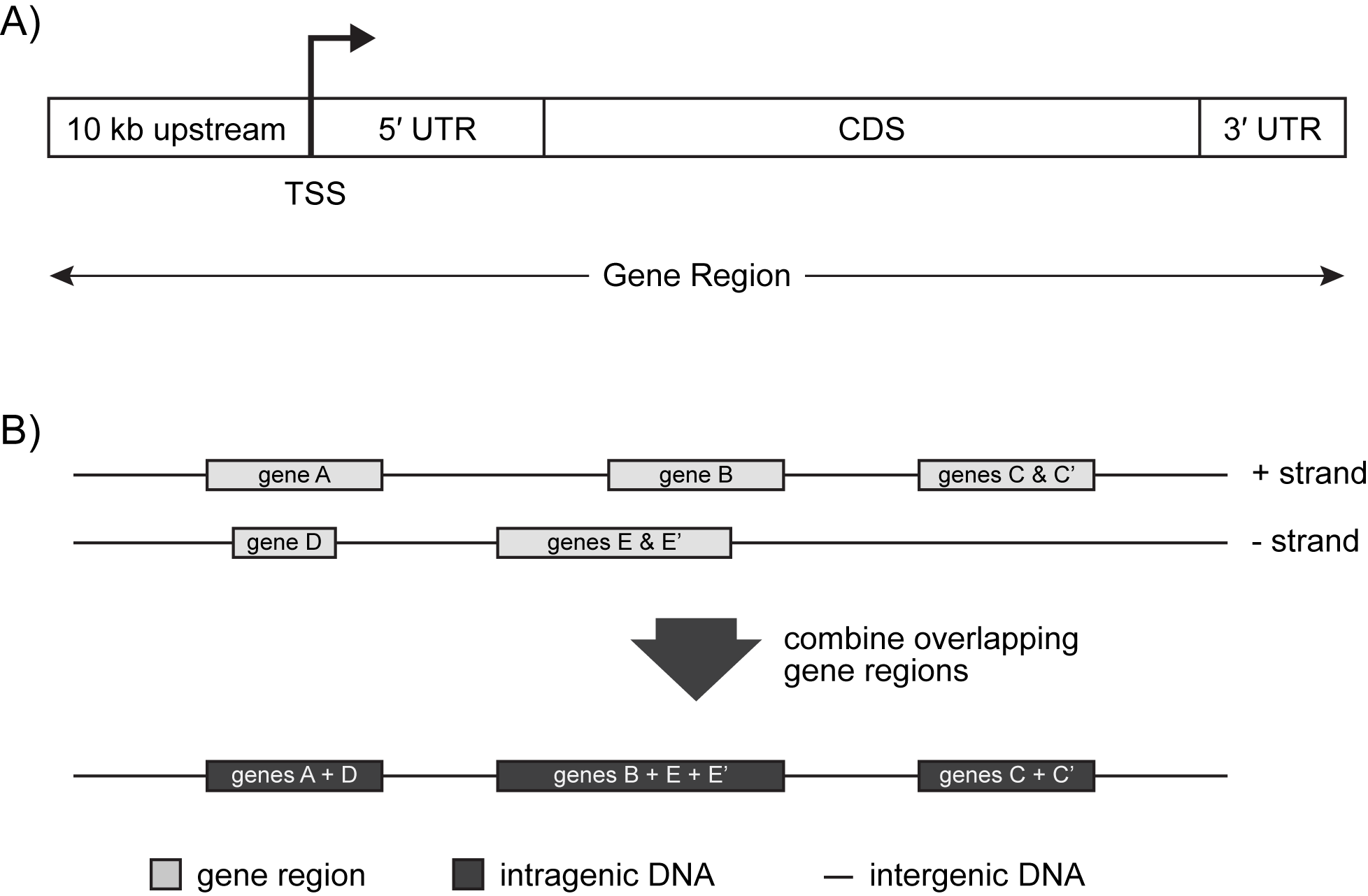

Supplement: Additional file 3 — Definitions of various genomic regions used to map regions of AhR enrichment. A) Genomic locations from the UCSC Genome Browser refGene database were used to obtain sequences for 10 kb region upstream of the TSS, the 5' and 3' UTRs, and the CDS of every known human, mouse and rat RefSeq sequence. A gene region is defined as the sequence spanning the region 10 kb upstream of a TSS through to the end of the 3' UTR. B) Intragenic DNA regions in a genome were determined by combining the non-overlapping gene regions. For example, gene regions of tissue specific isoforms of a gene that have different TSS positions were merged to determine the longest spanning range (genes C & C' and genes E & E'). Additionally, overlapping genes on both strands of the genome were also merged (genes B + E + E'). Non-transcribed DNA segments that span the regions between adjacent intragenic regions are defined as the intergenic DNA regions. [file 1471-2164-12-365-S3.PNG]

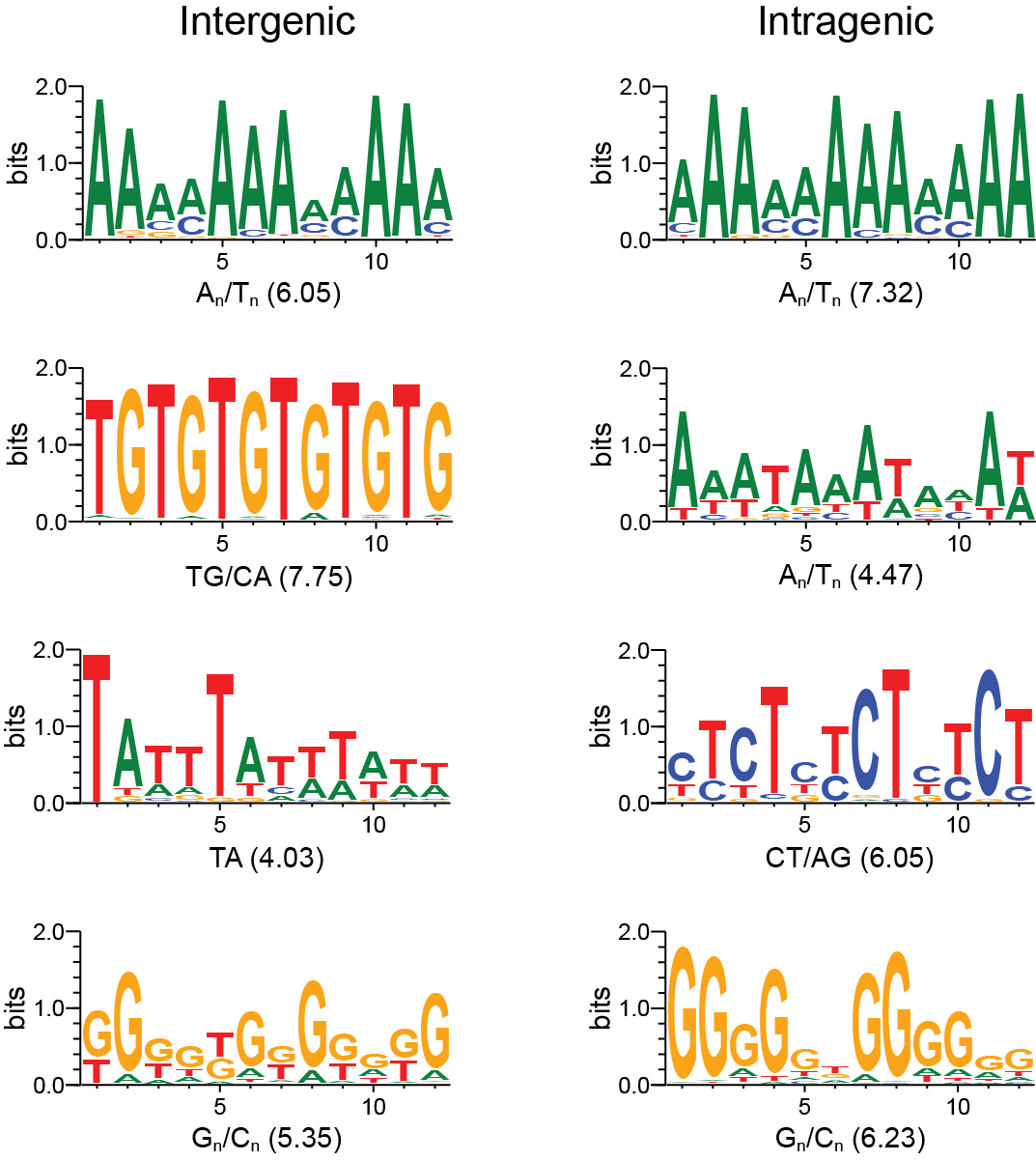

Supplement: Additional file 9 — Repetitive sequence elements identified in the de novo motif analysis of significant intragenic and intergenic AhR enriched regions (FDR < 0.01) lacking a DRE core. The repetitive over-represented motifs from each region are shown with their consensus and reverse complement sequence, and the Gibbs motif sampler score. [file 1471-2164-12-365-S9.PNG]
