# Supplementary material for: Integration of Genome-Wide Computation DRE Search, AhR ChIP-chip and Gene Expression Analyses of TCDD-Elicited Responses in the Mouse Liver
Source: BMC Genomics. 2011 Jul 15;12:365. doi: 10.1186/1471-2164-12-365 (PMC3160422; doi:10.1186/1471-2164-12-365)
Supplement: Additional file 12 — Circos plots integrating DRE analysis, AhR enrichment (2 hrs; FDR < 0.01) and heatmaps for hepatic differential gene expression responses (|fold change| ≥ 1.5 and P1(t) > 0.999) induced by TCDD across the genome. Circos plots illustrate the ideograms for each individual chromosome and the entire genome and integrate the results of the DRE, ChIP-chip and gene expression analyses. [file 1471-2164-12-365-S12.PDF]

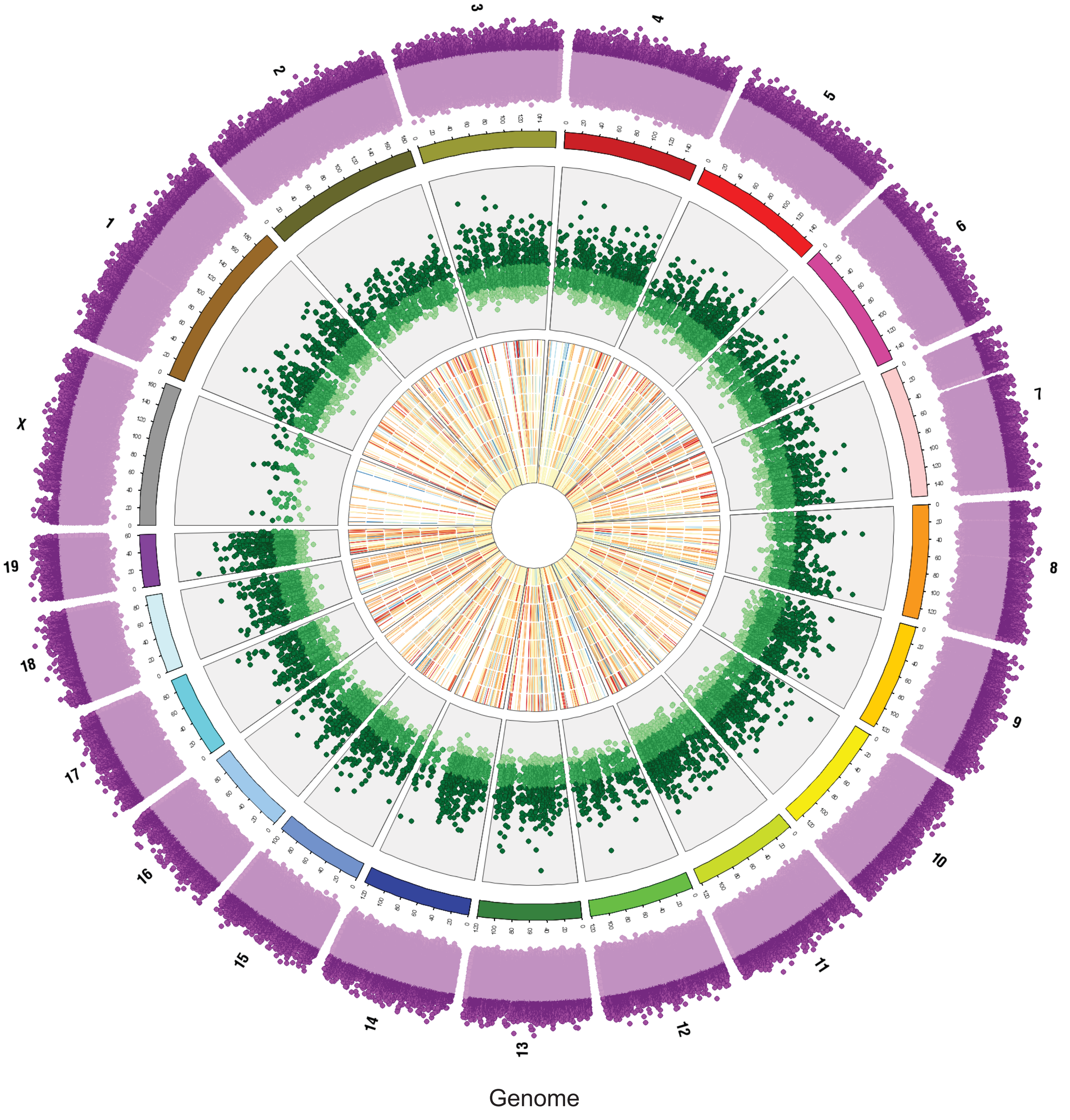
1
2
3
4
5
6
7
8
9
10
11
12
13
14
15
16
17
18
19
X
Genome

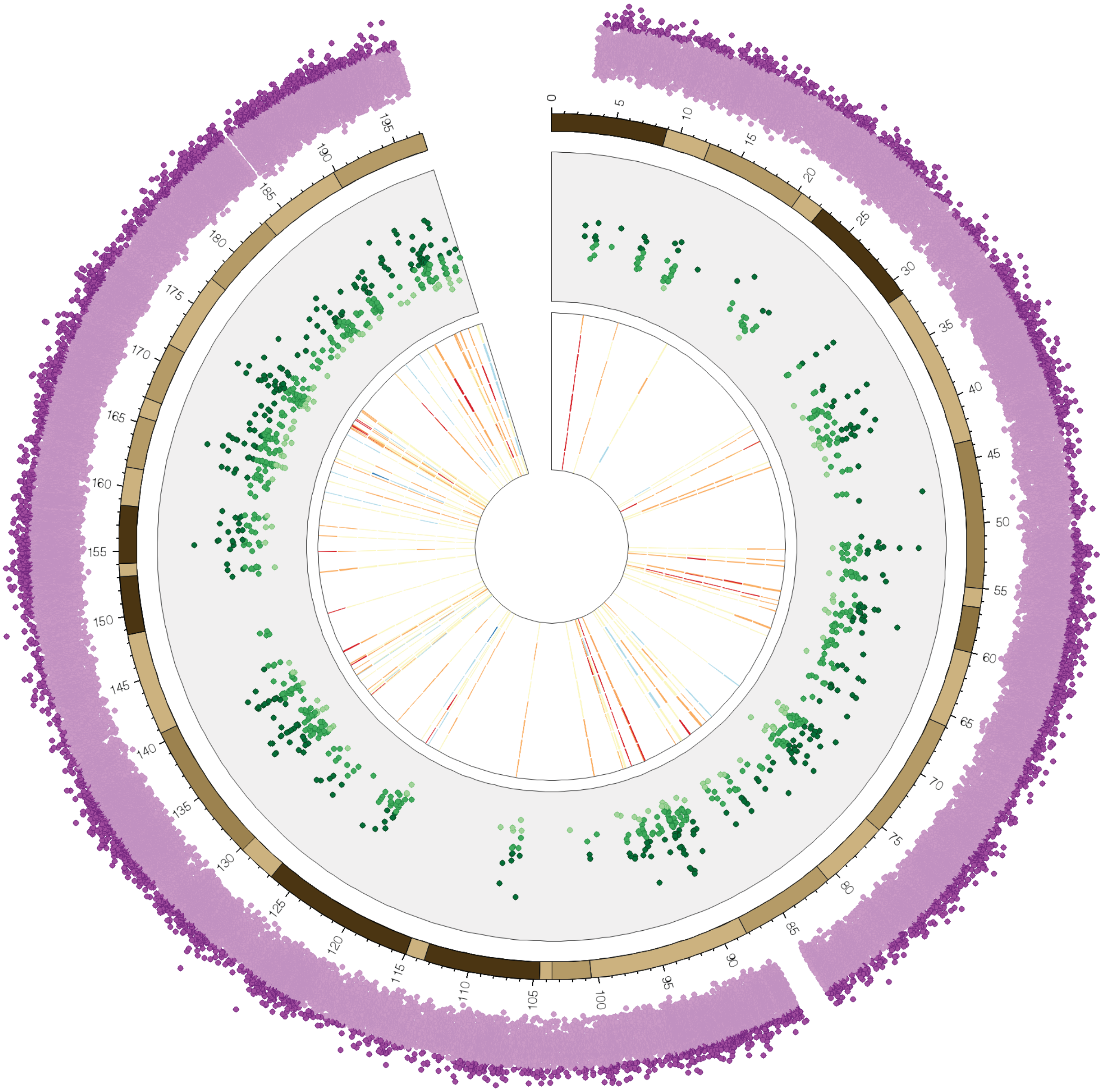

Chromosome 1

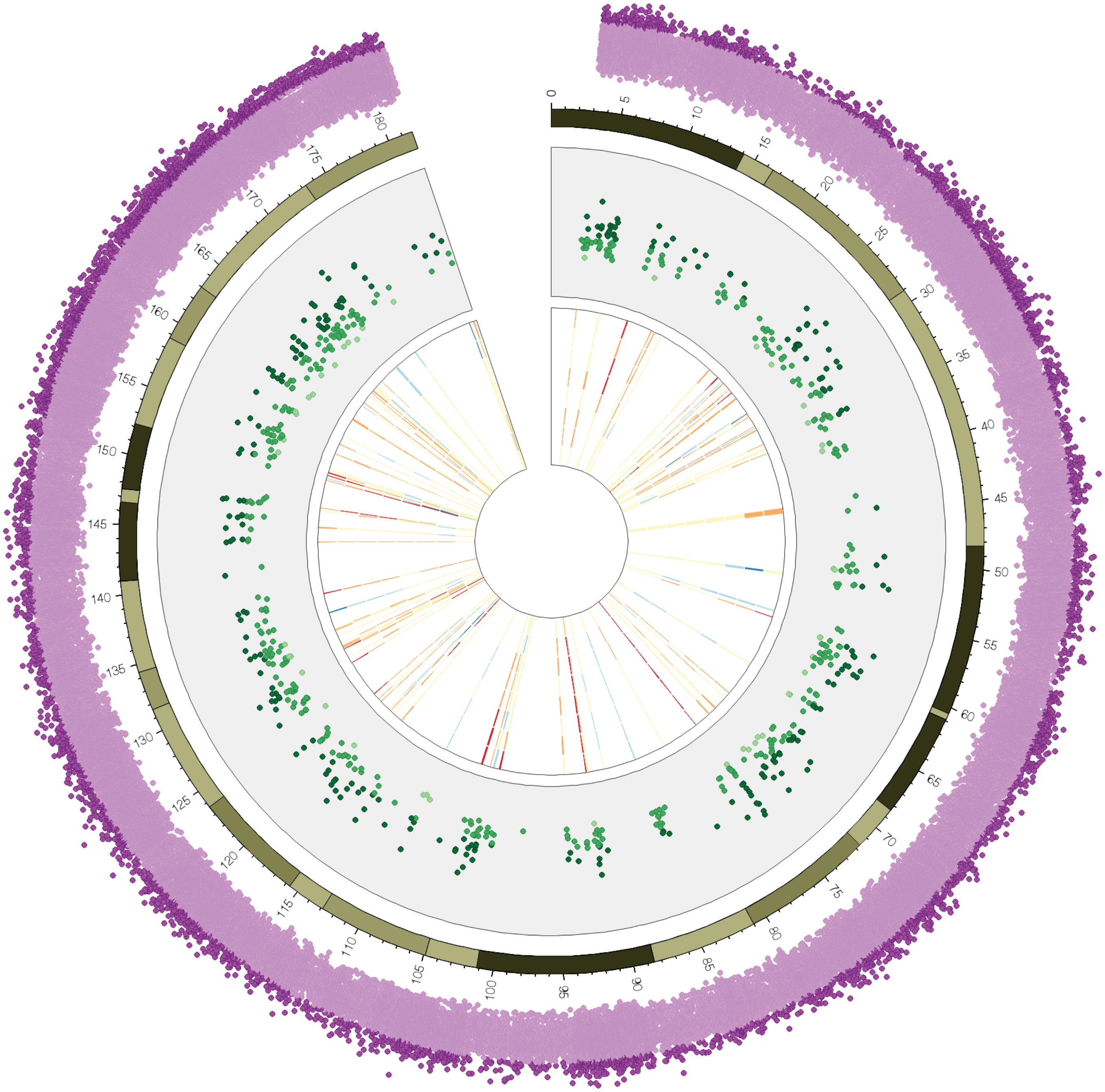

Chromosome 2

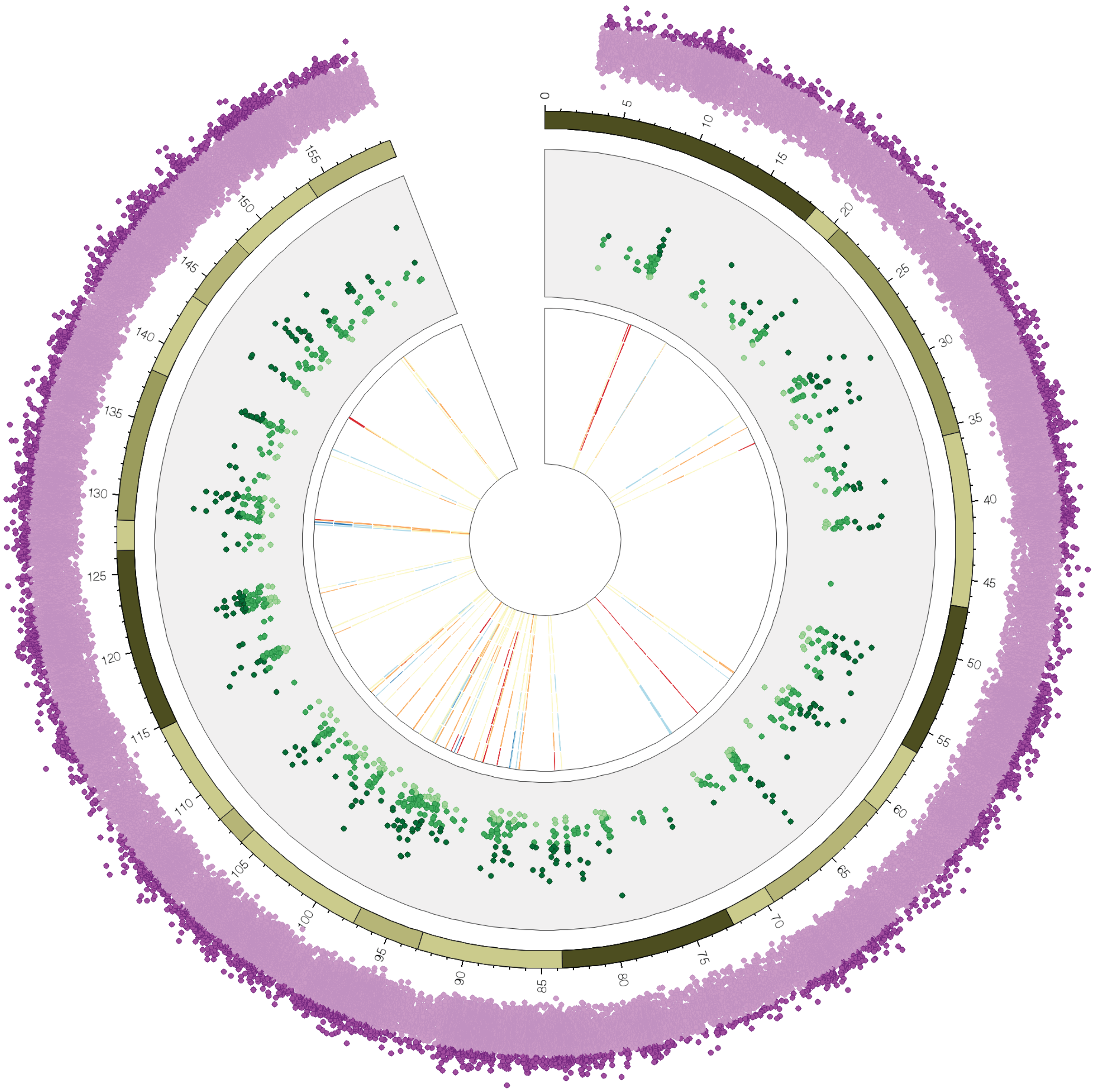

Chromosome 3

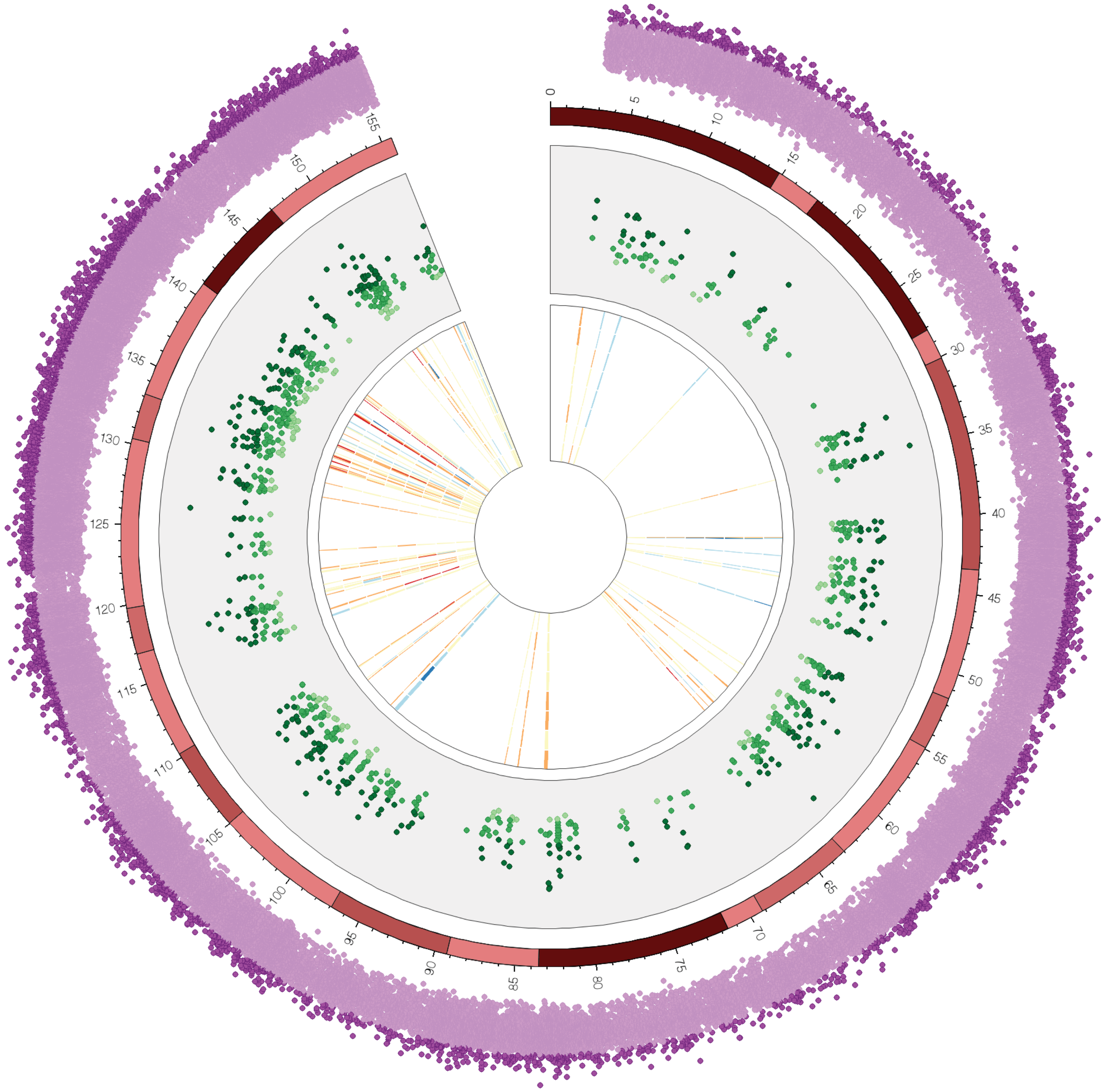

Chromosome 4

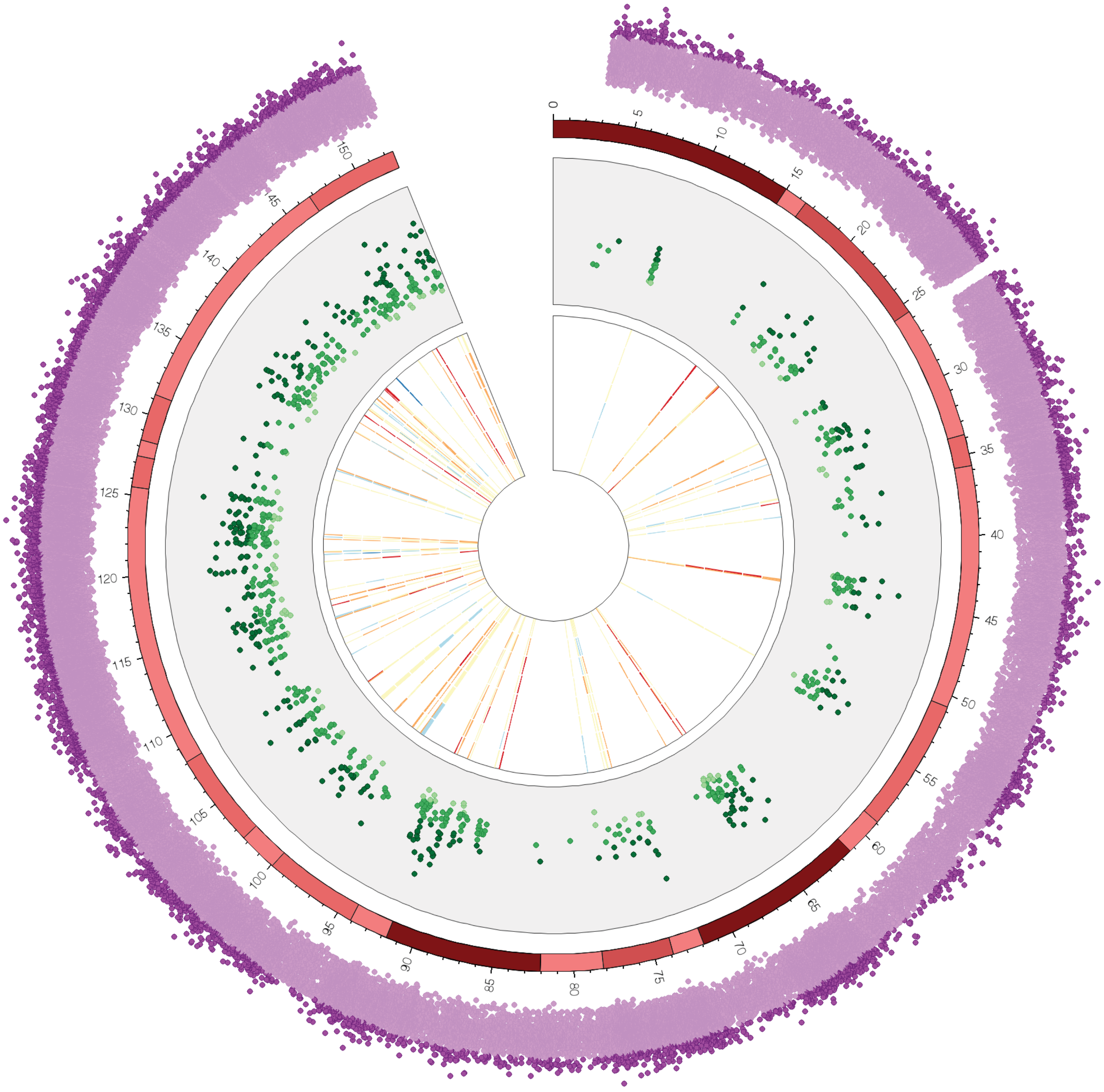

Chromosome 5

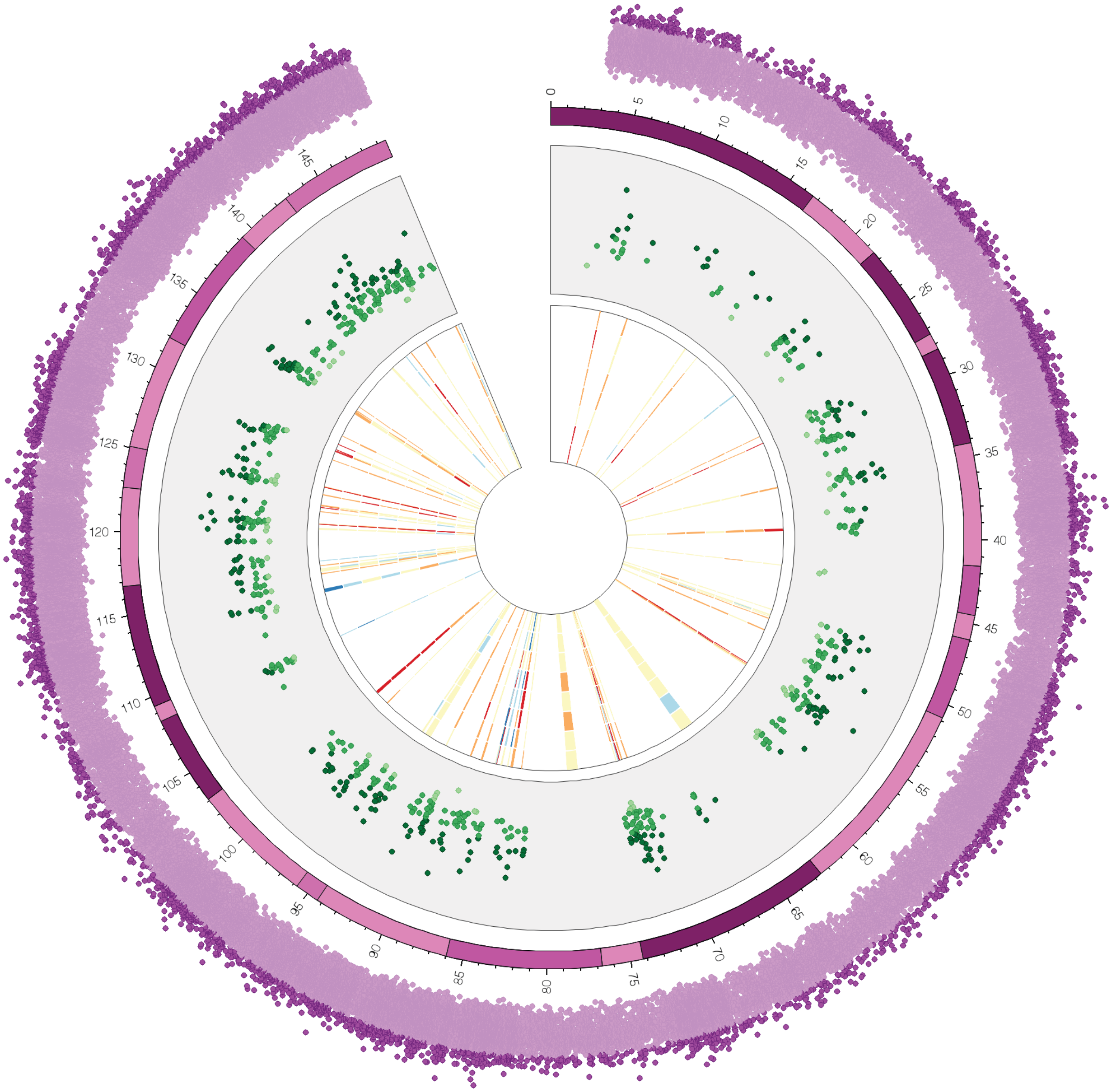

Chromosome 6

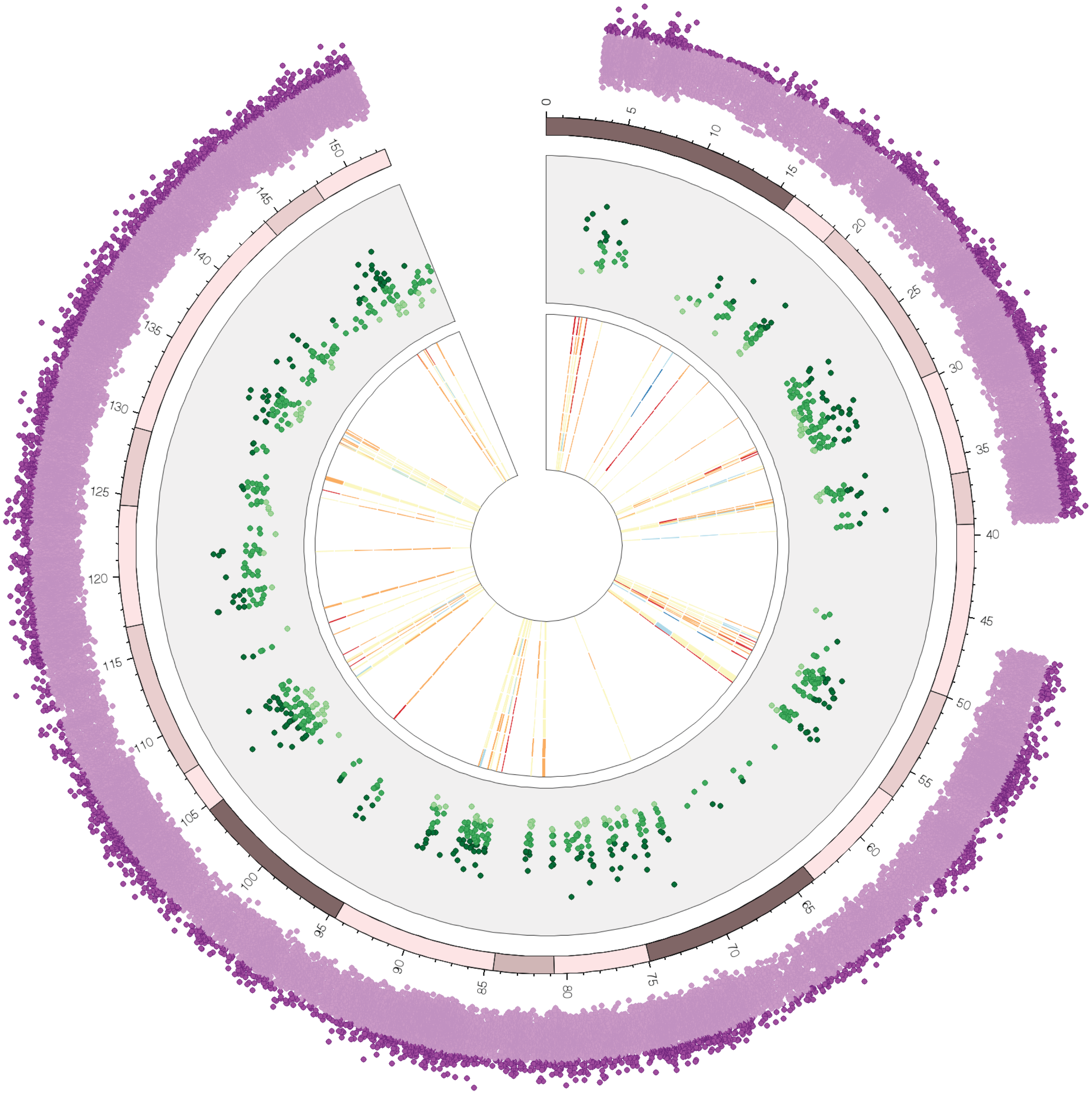

Chromosome 7

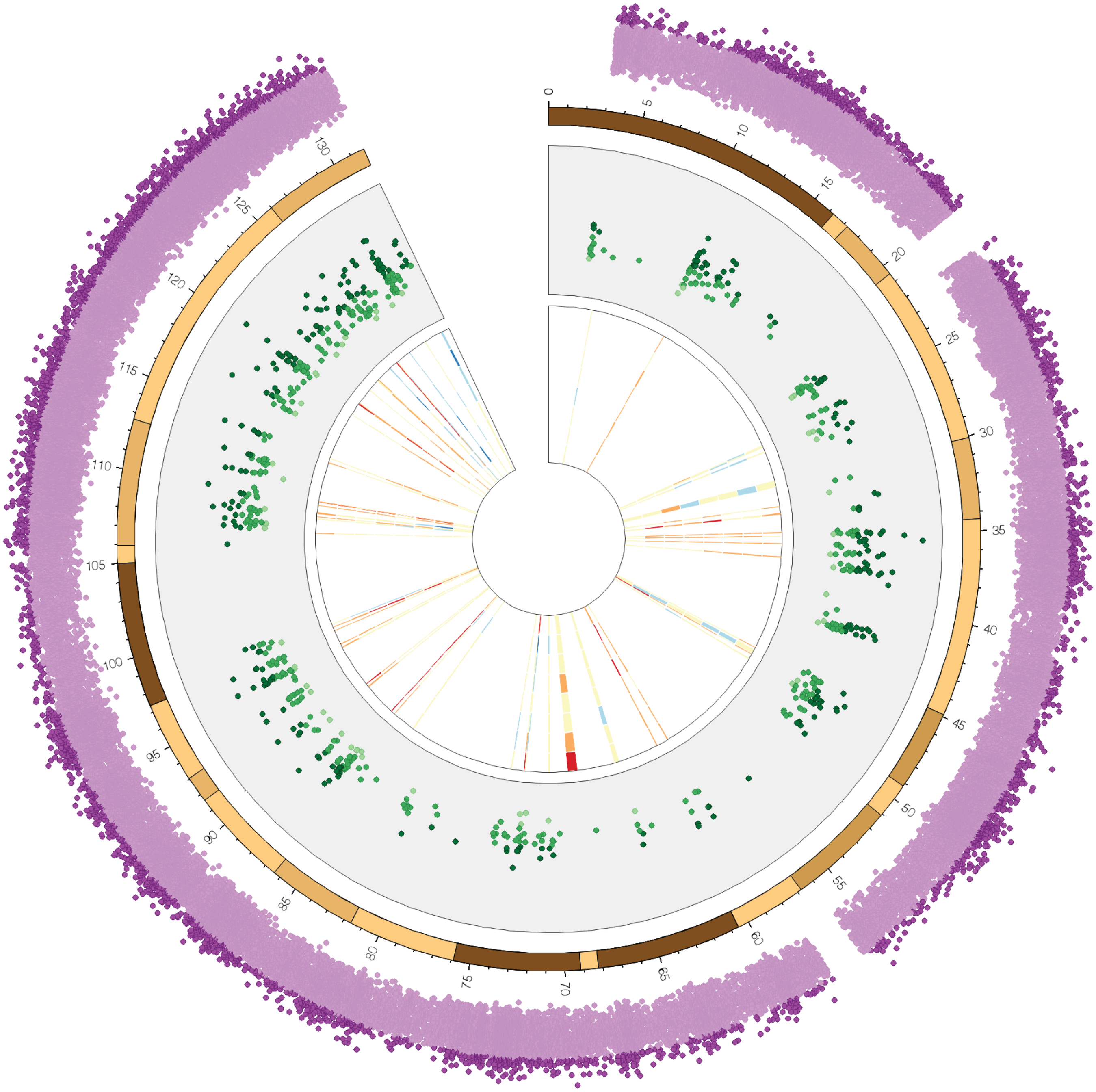

Chromosome 8

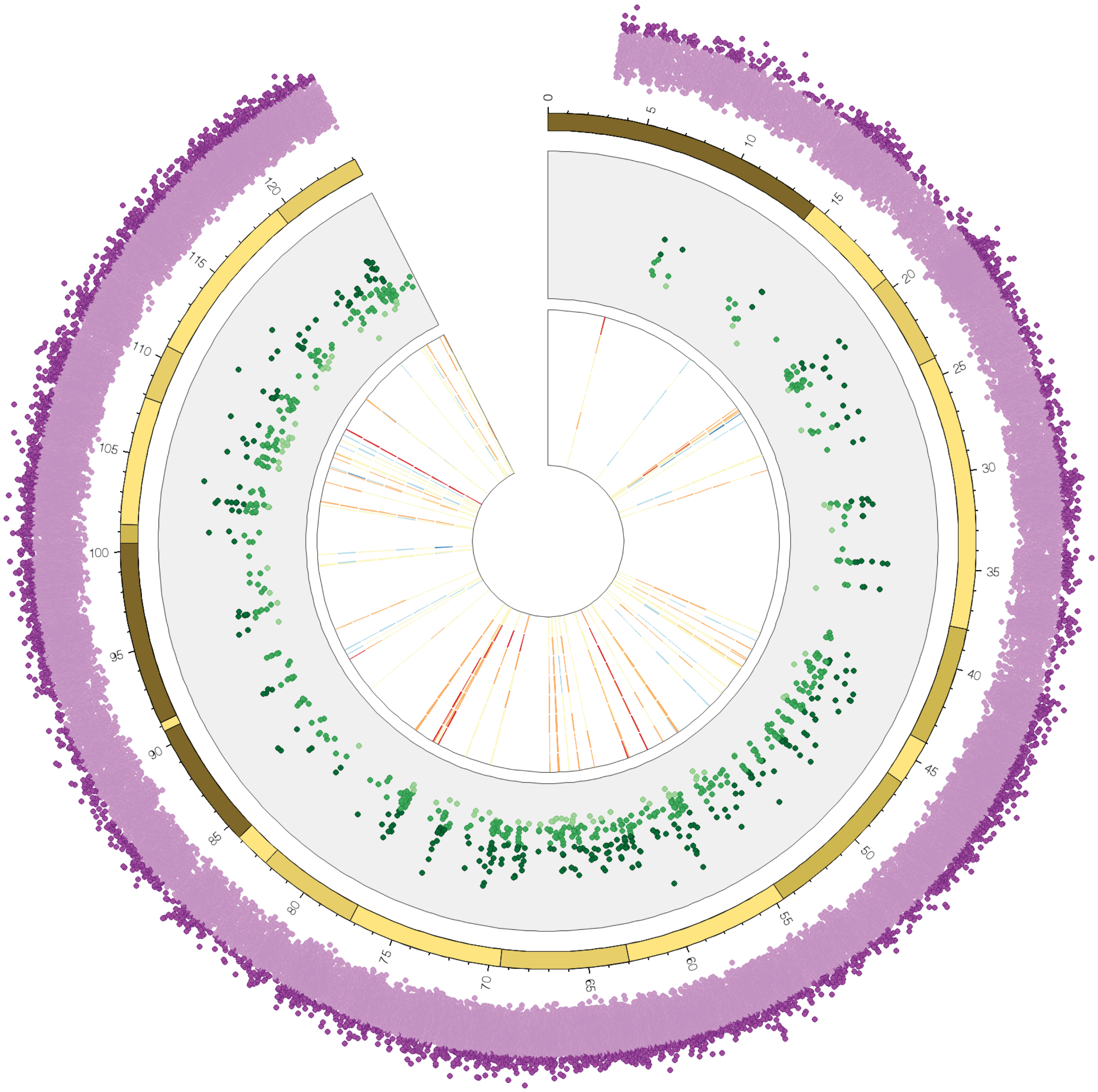

Chromosome 9

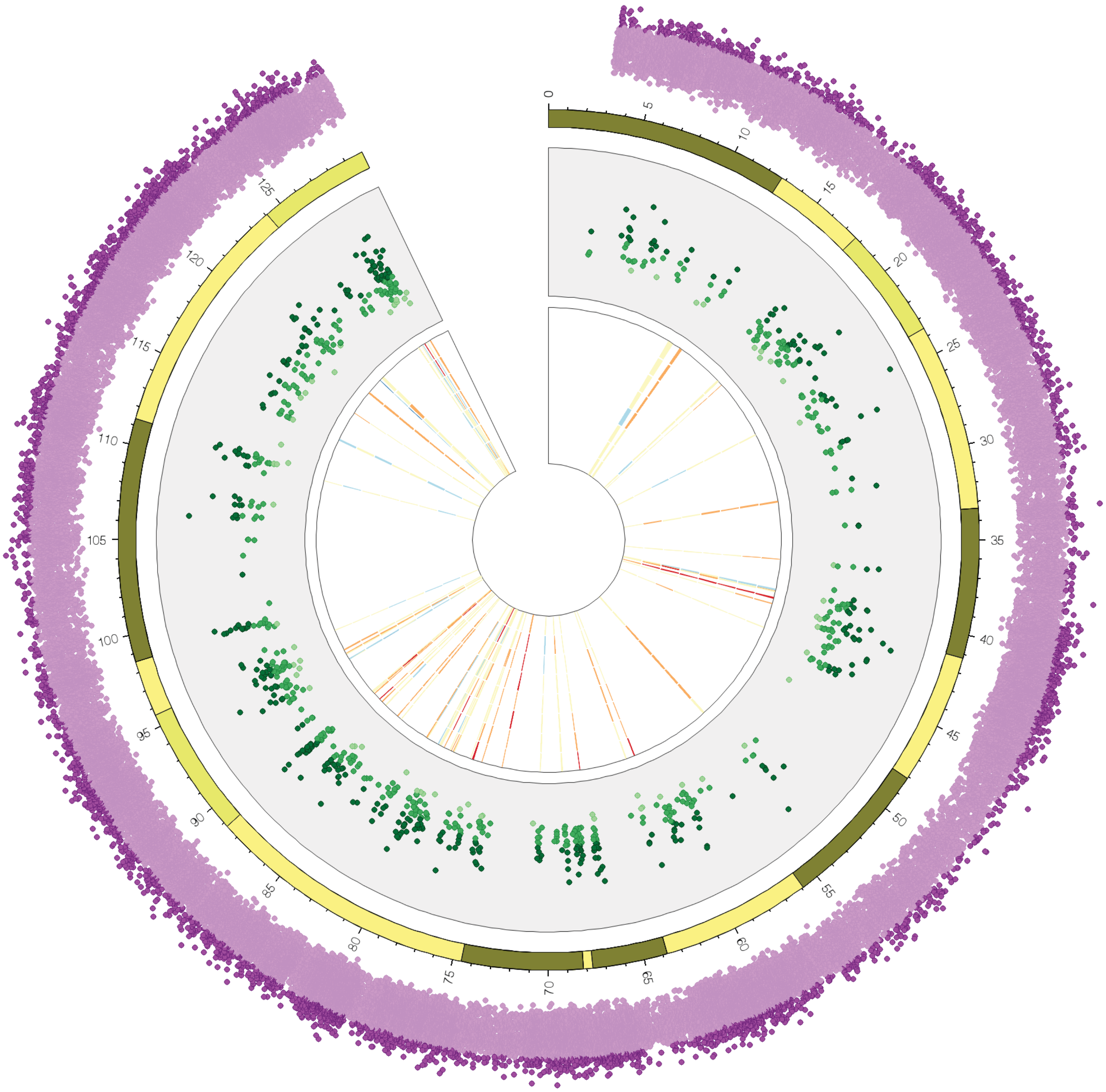

Chromosome 10

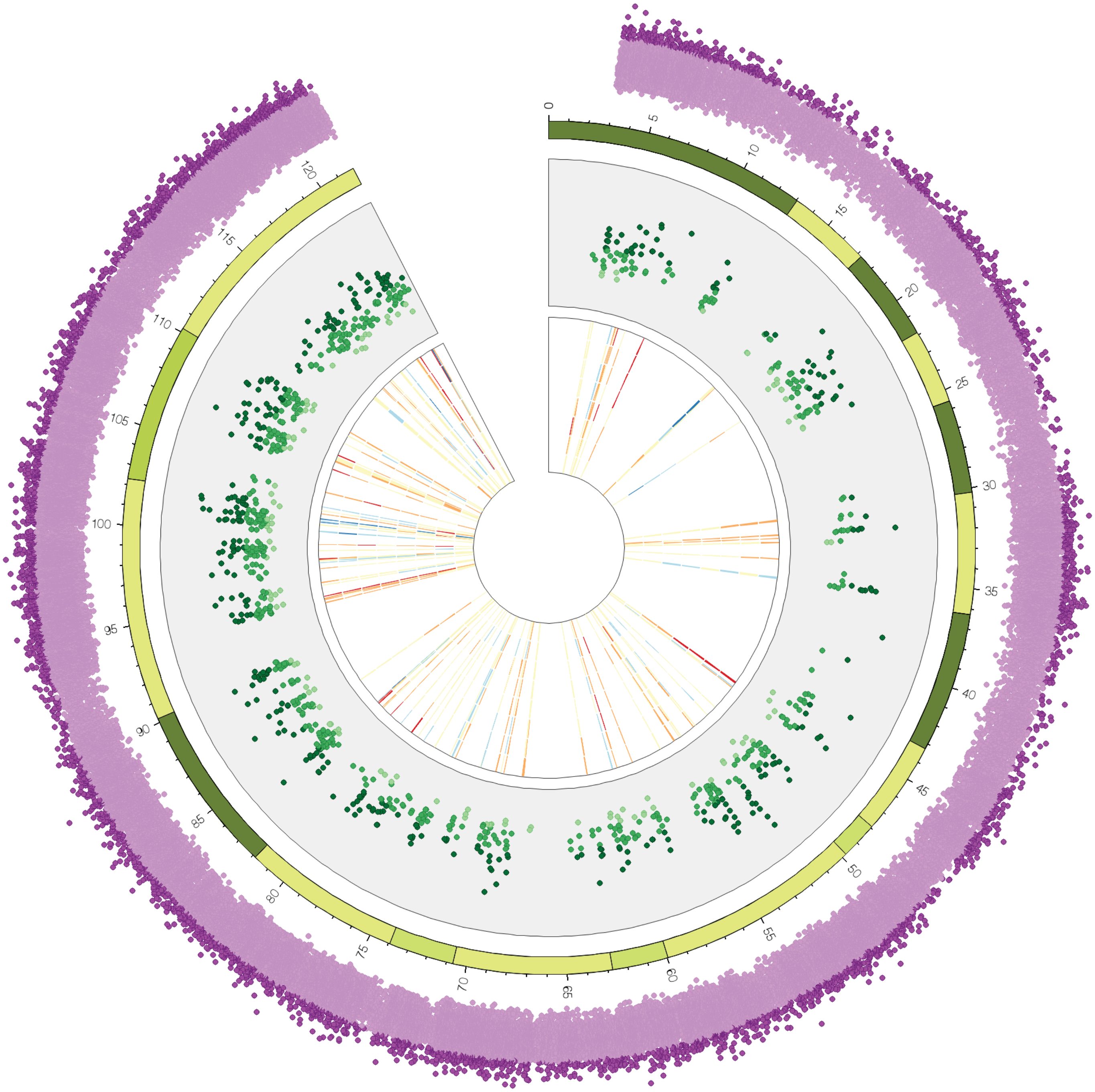

Chromosome 11

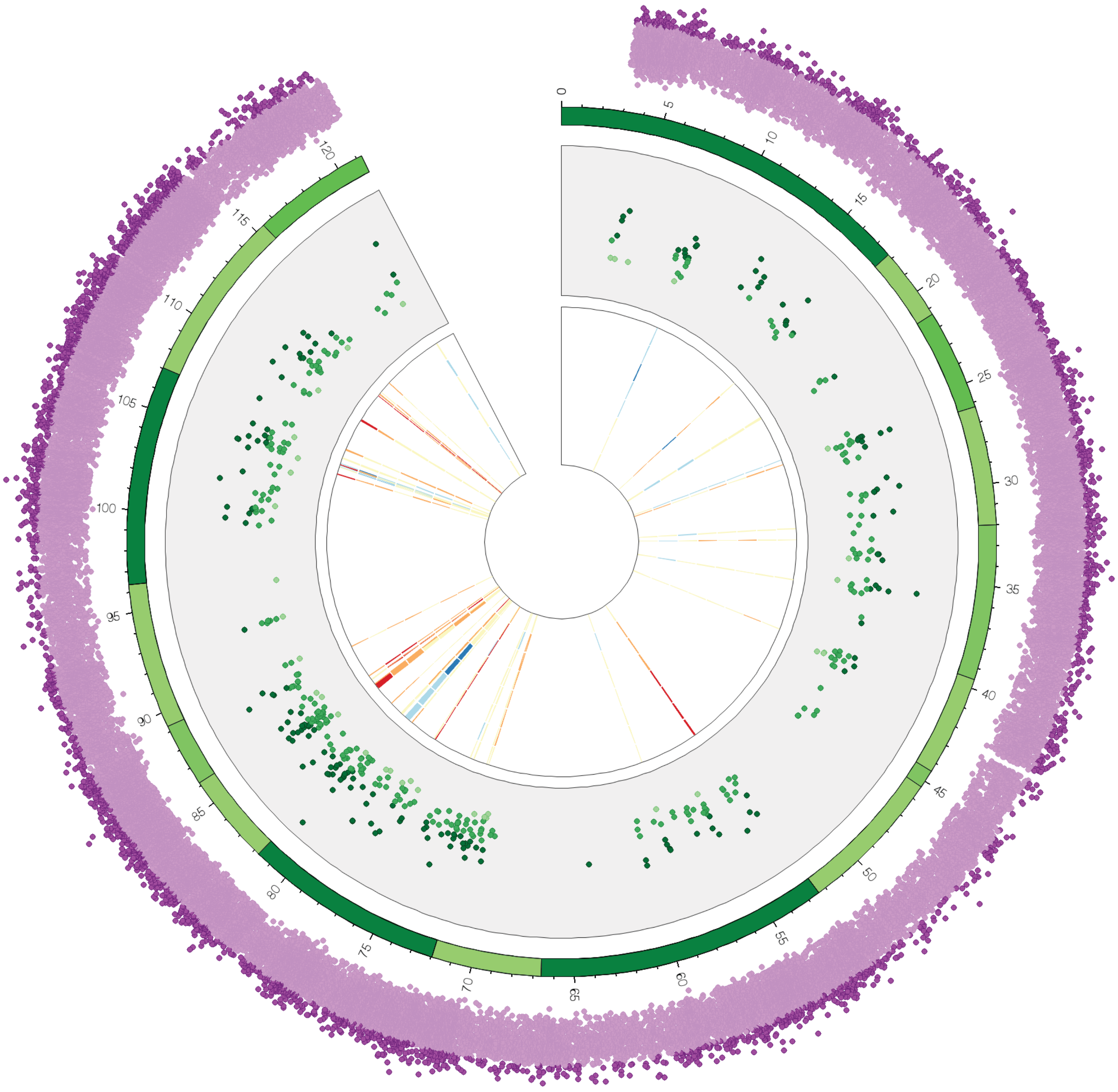

Chromosome 12

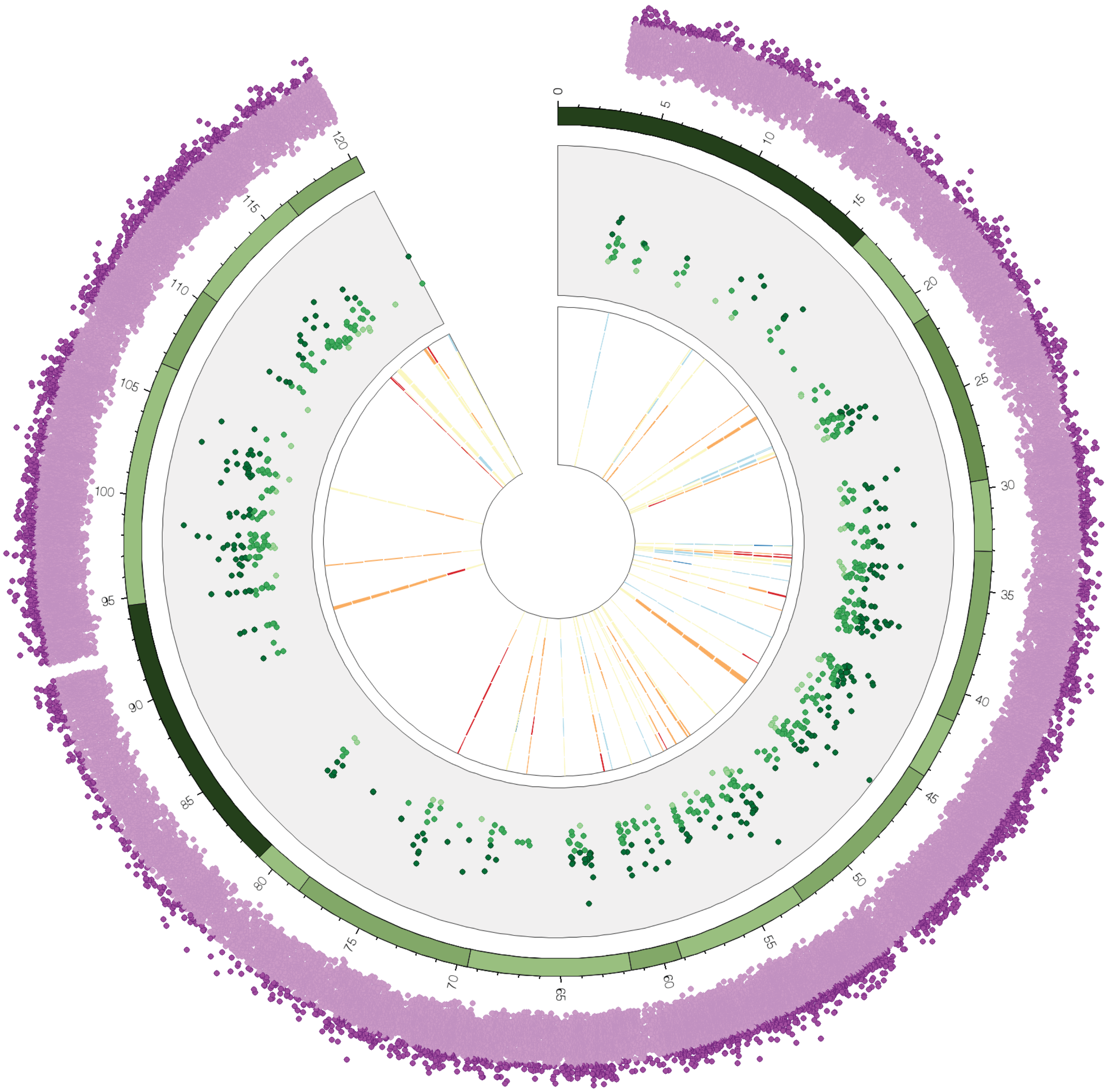

Chromosome 13

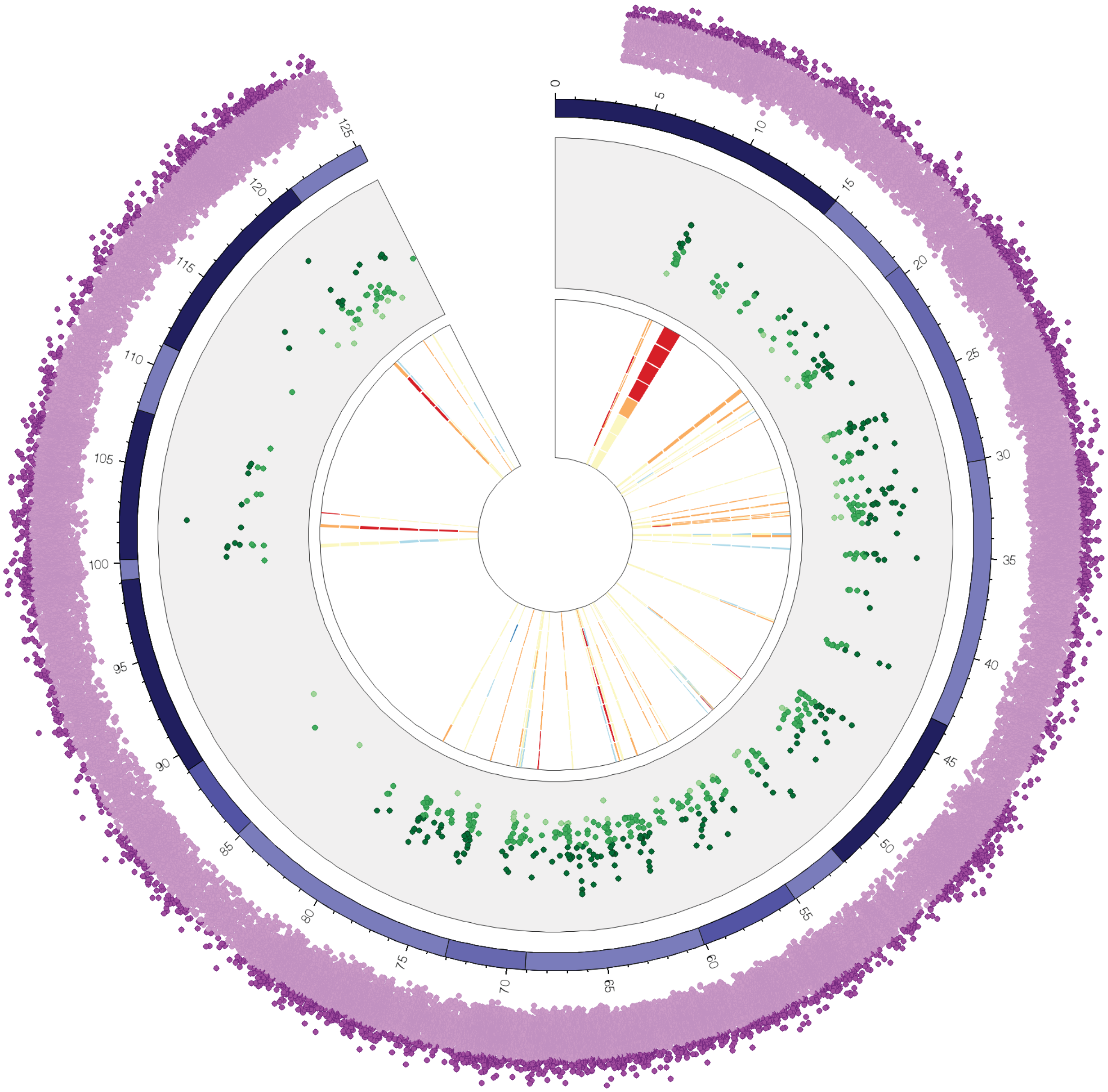

Chromosome 14

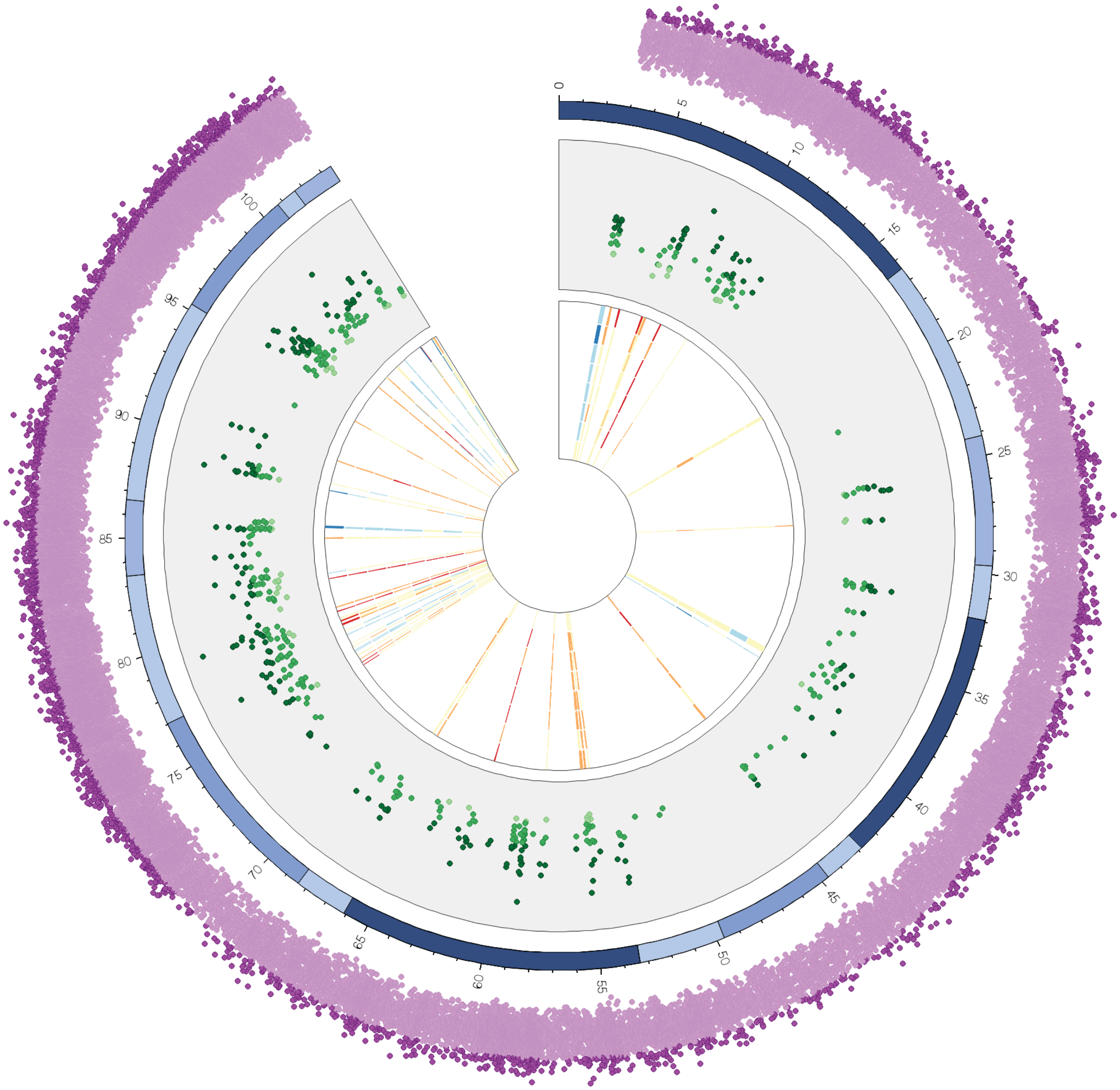

Chromosome 15

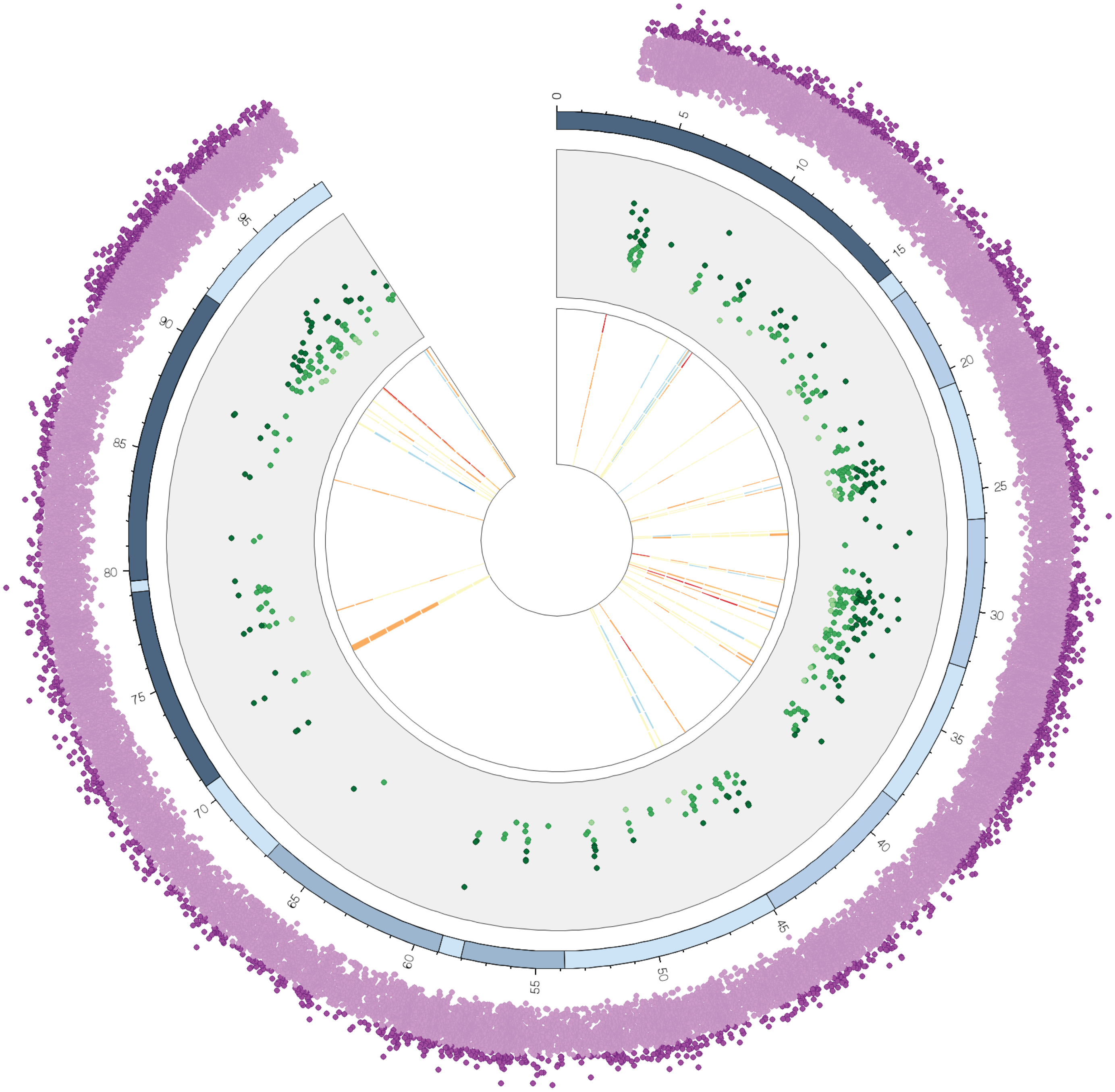

Chromosome 16

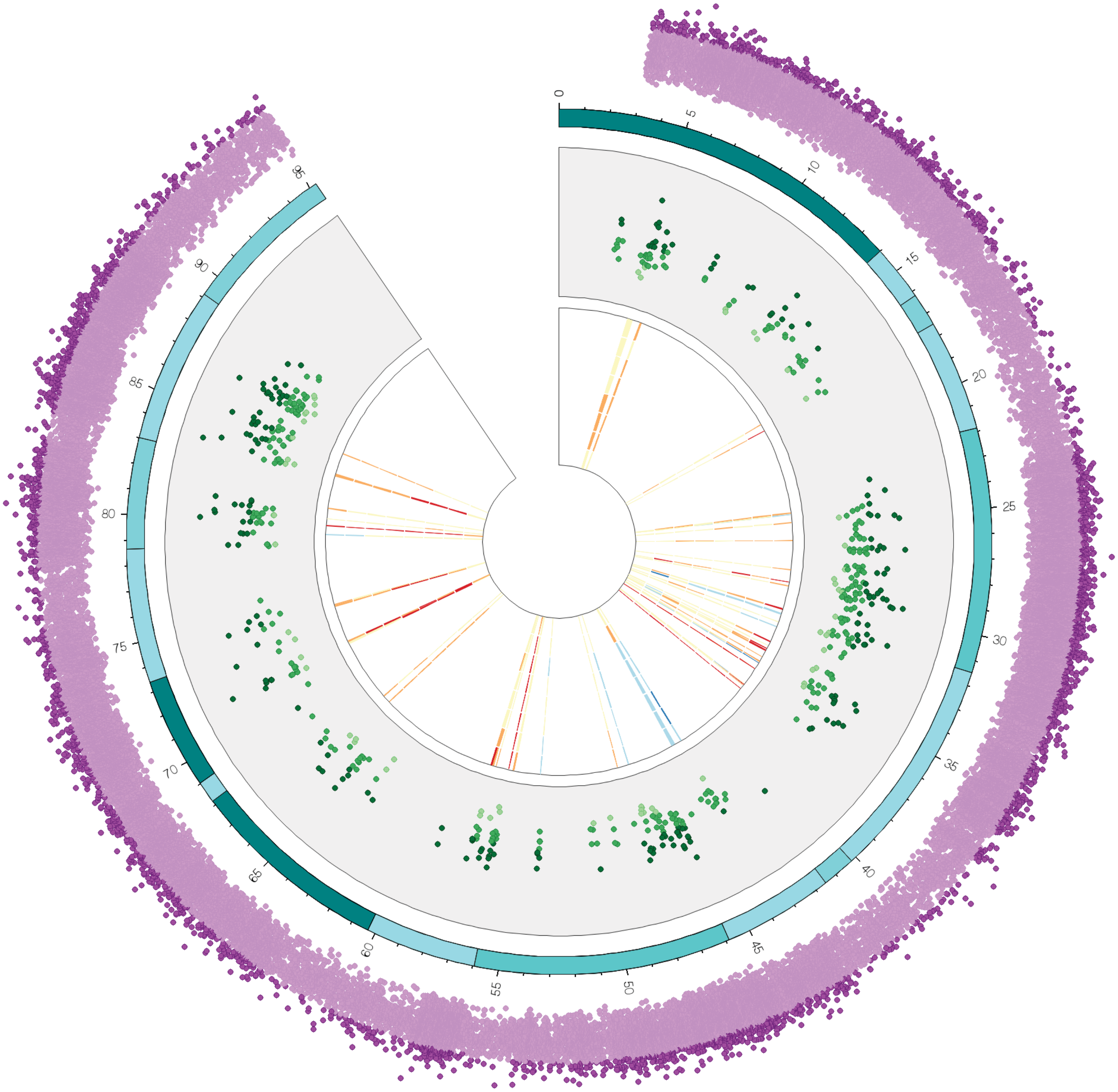

Chromosome 17

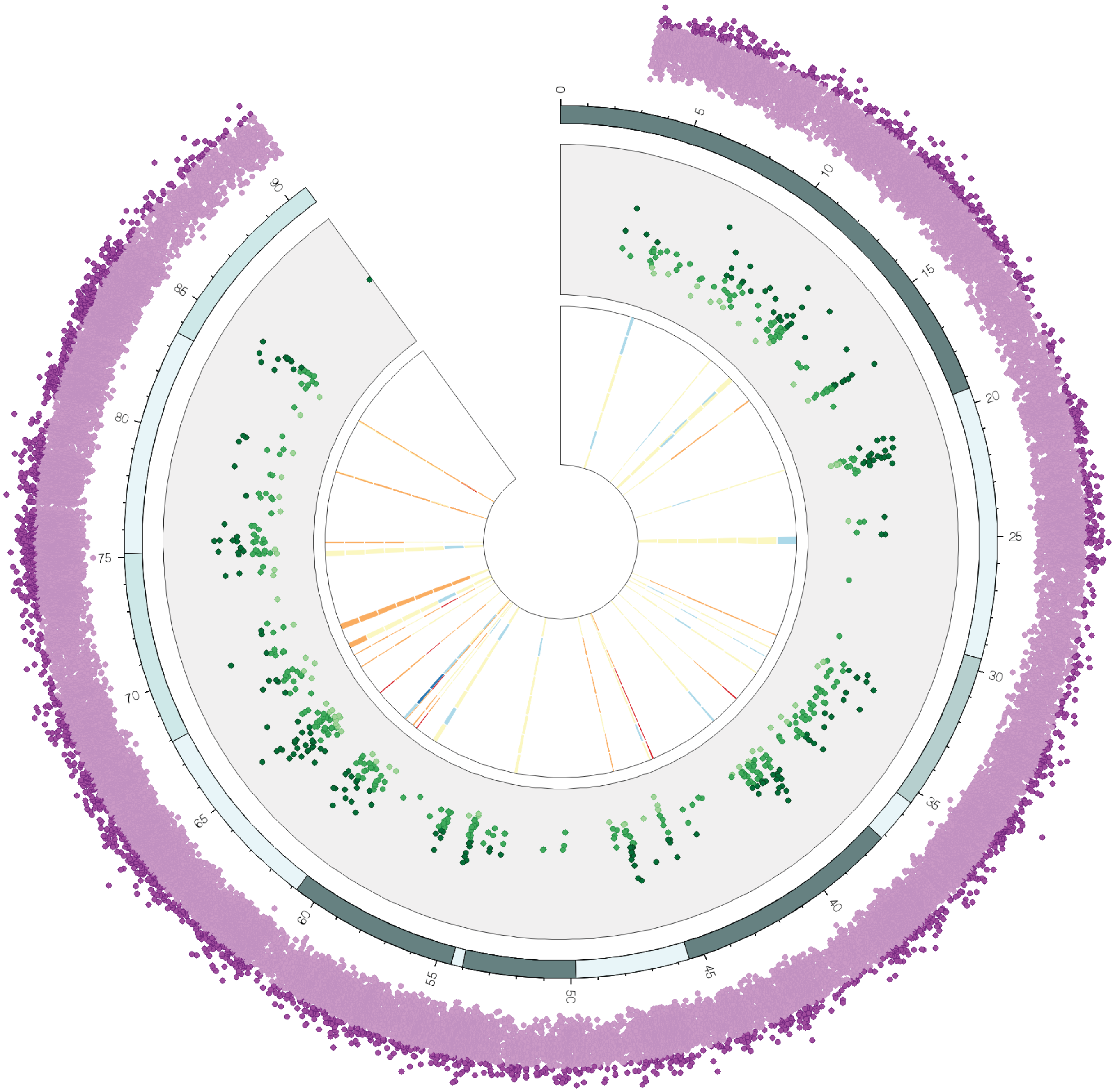

Chromosome 18

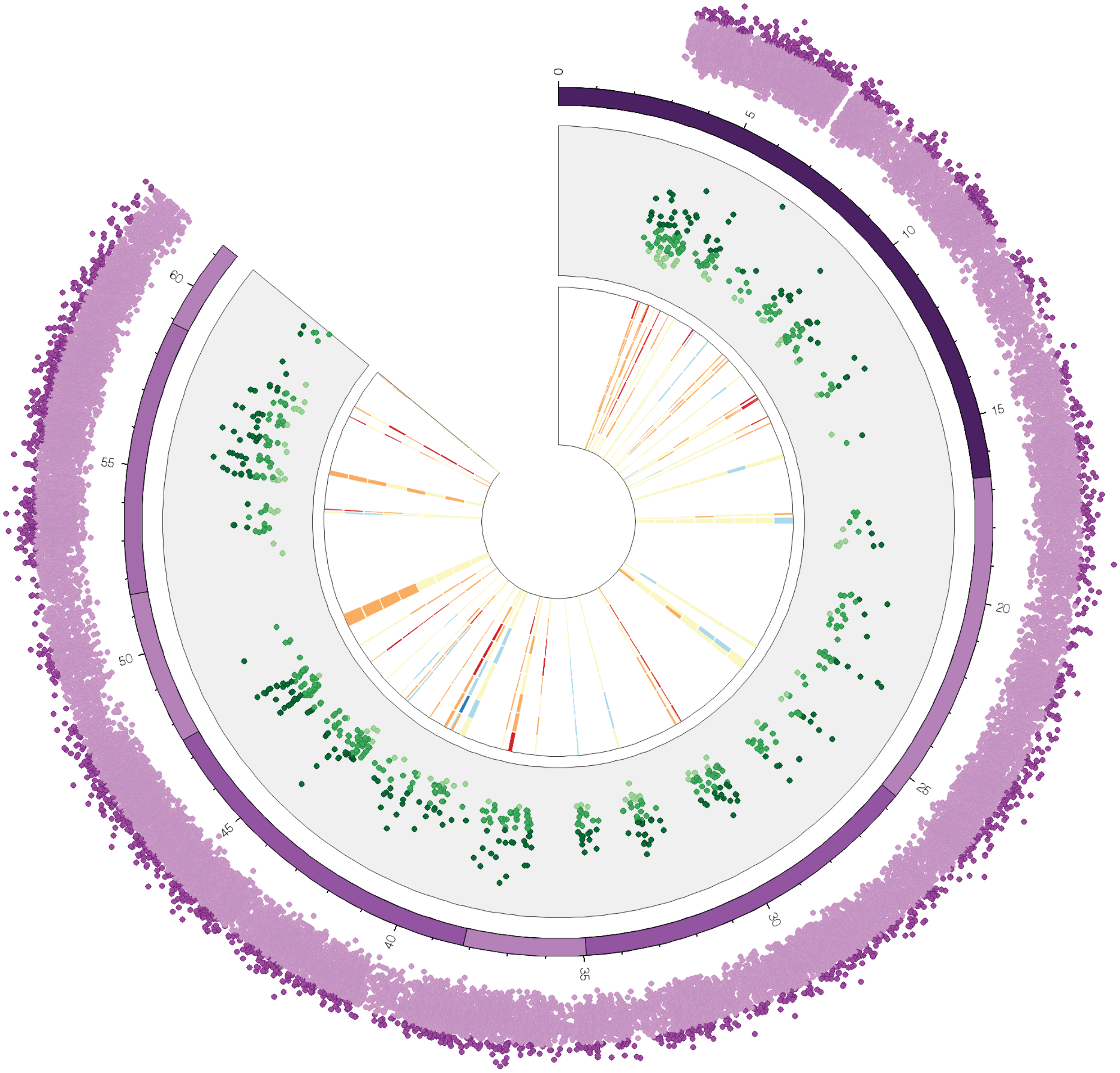

Chromosome 19

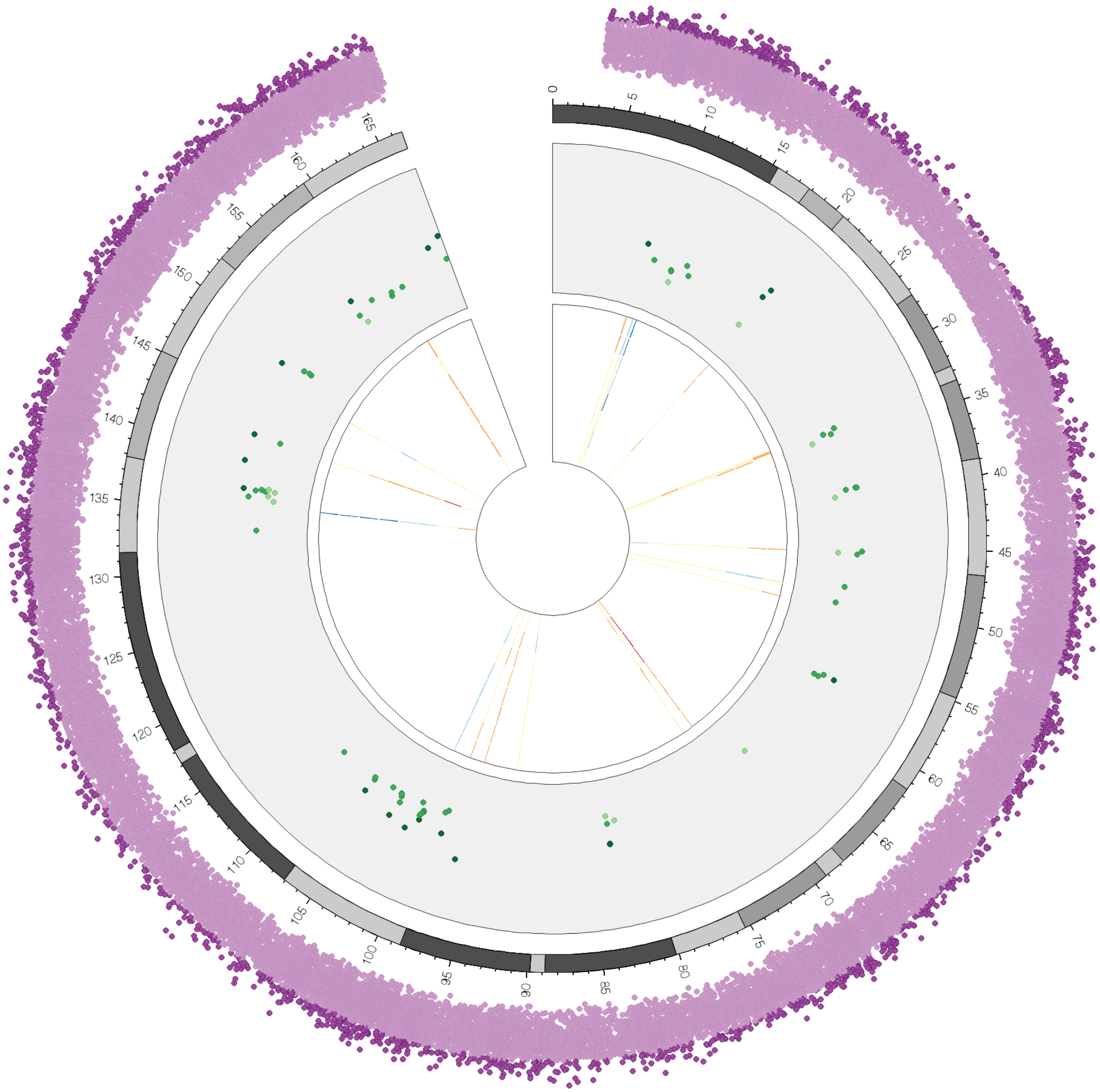

Chromosome X
